# Limonoids From the Genus *Melia* (Meliaceae): Phytochemistry, Synthesis, Bioactivities, Pharmacokinetics, and Toxicology

**DOI:** 10.3389/fphar.2021.795565

**Published:** 2022-01-24

**Authors:** Wenxiang Fan, Linhong Fan, Zhengtao Wang, Li Yang

**Affiliations:** ^1^ The MOE Key Laboratory of Standardization of Chinese Medicines, Shanghai Key Laboratory of Compound Chinese Medicines, and SATCM Key Laboratory of New Resources and Quality Evaluation of Chinese Medicines, Institute of Chinese Materia Medica, Shanghai University of Traditional Chinese Medicine, Shanghai, China; ^2^ Shanghai Frontiers Science Center of TCM Chemical Biology, Institute of Interdisciplinary Integrative Medicine Research, Shanghai University of Traditional Chinese Medicine, Shanghai, China

**Keywords:** limonoids, genus *Melia*, toosendanin, anti-tumor, insecticide, toxicology

## Abstract

Limonoids, as the vital bioactive chemical compounds in genus *Melia* plants, have attracted significant attention owing to their exclusive structural characteristics and remarkable biological activity. These compounds can be usually classified into two categories, including the ring-intact group and the ring-C-seco group. Benefiting from the development of separation and analysis technology, more than 200 limonoids have been isolated and identified from this genus. There is growing evidence that limonoids from genus *Melia* possess diverse pharmacological activities, especially anti-cancer effects, insecticidal activities, and anti-botulism effects. Toosendanin, one of the paramount limonoids, was considered as the pivotal bioactive marker in two medicinal herbs, including *Melia toosendan* Sieb. et Zucc and *Melia azedarach* L. In particular, limonoids are found to exhibit non-negligible toxic effects, a finding which needs further research. Besides this, the lack of clinical research data seriously hinders its further development and utilization, and necessary clinical trials should be taken into consideration. In this review, we systematically summarized the phytochemical compounds and their synthesis methods, pharmacological activities, and the structure–activity relationship, pharmacokinetics, and toxicology of genus *Melia*-derived limonoids. We believe that this up-to-date review could provide scientific evidence for the application of limonoids as agents beneficial to health in future clinical practice.

## Introduction

Genus *Melia*, a model genus of Meliaceae, has about 20 species in the world and is widely distributed in tropical and subtropical regions of the Eastern Hemisphere. Among them, *Melia toosendan* Sieb. et Zucc., *Melia azedarach* L., *Melia azedarach* var. *japonica*, and *Melia volkensii* Gürke have received a lot of attention (Liu et al., 2010). It is worth noting that *Azadirachta indica* A. Juss., a plant of the genus *Azadirachta*, is often wrongly recognized as *M. azedarach* L. for their similar morphological characteristics and botanical name (Saleem et al., 2018). Owing to their multiple bioactivities, the *Melia* plants have been used as folk herbs in treating leprosy, eczema, asthma, malaria, fever, and pain (Xie et al., 2008). *M. toosendan* Sieb. et Zucc and *M. azedarach* L., two common medicinal plants in China, have been used to treat diseases for thousands of years. The first traditional usage can be traced back to *Shen Nong Ben Cao Jing*, which is the earliest medical monograph in China that was written during the Eastern Han Dynasty (AD 25–220). In this monograph, the two herbs functioned as treatment for anxiety, destroying parasites, and promoting diuresis. According to *Ben Cao Gang Mu* (AD 1578), which is another famous medical classic, *M. toosendan* and *M. azedarach* could treat stomach ache and hernia. In addition, the ability to clear heat and promote diuresis was reported in *Ben Cao Jing Shu* (AD 1625). According to Chinese Pharmacopoeia, *M. toosendan* and *M. azedarach* have been recorded as insecticide and painkiller (Chinese Pharmacopoeia Commission, 2020). Various types of chemical compounds have been isolated and identified from different parts of genus *Melia* plants, including limonoids (triterpenoids), steroids, alkaloids, flavonoids, anthraquinones, *etc.* (Zhao et al., 2010). Modern pharmacological research demonstrated that limonoids, which are abundant in *Melia* species, exhibit a potential activity (Taylor, 1984). The word “limonoids” originated from the bitterness of lemon or other citrus fruits. Early chemical research of such compounds in Meliaceae started in 1960. The first limonoid compound named gedunin was isolated from wood of the West African plant *Entandrophragma angolense*, and its chemical structure was identified by comparison with limonin (Goerlich, 1960). The fundamental structure of limonoids is formed by the loss of four terminal carbons of the side chain in the apotirucallane or apoeuphane skeleton and then cyclized to form the 17β-furan ring, and thus limonoids are also known as tetranortriterpenoids (Tan and Luo, 2011). In this paper, a literature survey was carried out by searching the keywords including “limonoids”, “*Melia*”, “*Melia toosendan* Sieb*.* et Zucc”, and “*Melia azedarach* L.” from Pubmed, SciFinder, Science Direct, Scopus, the Web of Science, Google Scholar, China National Knowledge Infrastructure, and classic books of herbal medicine. All data were searched up to May 2021 to identify eligible studies. Some previous reviews have summarized the limonoids-related research progresses—for instance, the chemical compounds of *Melia* species and their bioactivities were summarized in 2010 (Zhao et al., 2010). In addition, a fantastic review has comprehensively covered the limonoids from Meliaceae, and their pharmacological effects were also concluded (Tan and Luo, 2011). Nevertheless, our work attempts to offer some constructive information that is favorable to the development of genus *Melia*-derived limonoids that originated from traditional medicinal herbs. Herein we systematically summarize the phytochemistry, synthesis, pharmacological activities, structure–activity relationships, pharmacokinetics, and safety aspects of limonoids, hoping that these could propel forward the exploration for this kind of valuable compound. Furthermore, the future research perspectives and difficulties are discussed as well.

## Chemical Compounds

**FIGURE 1:**
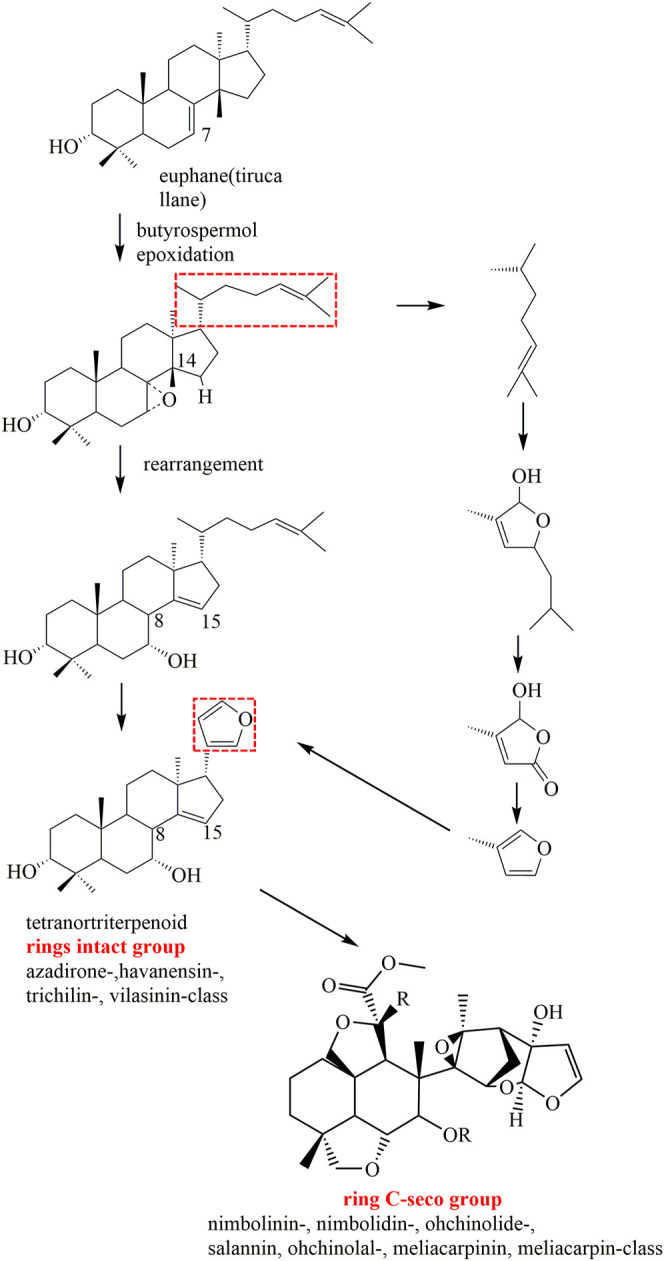
Proposed major biosynthesis pathways of limonoids in genus *Melia*.

After years of phytochemical research, more than 200 limonoids have been isolated and identified from genus *Melia* plants. As expected, the majority of these compounds originated from *M. toosendan* and *M. azedarach* because these two species are commonly used as medicinal herbs. In addition, the distribution of these compounds also varied in different parts of the plants, of which the fruit, bark, and root bark possessed higher content. Interestingly, those parts mentioned above are consistent with the medicinal parts of *M. toosendan* and *M. azedarach*. Therefore, isolation of bioactive compounds from medicinal herbs is a promising strategy for discovering lead compounds in drug development. At present, it is generally acknowledged that the precursors for the biogenic synthesis of limonoids are two types of tetracyclic triterpenoids, including tirucallane and euphane. The biosynthesis pathways of limonoids are shown in Figure1. Firstly, the Δ^7^-double bond is oxidized to 7-epoxy, which was subsequently opened to cause a Wagner–Meerwein shift of Me-14 to C-8, and finally leads to the formation of OH-7 and the introduction of a double bond at C-14/15. On this basis, four carbons are lost from the rear side chain to form a 17-β-furan ring, and the last step is finished after the formation of the 4,4,8-trimethyl-steroid skeleton, which was regarded as the basic limonoid skeleton (Tan and Luo, 2011). The limonoids in genus *Melia* are mainly classified into two classes, including the ring-intact limonoids and the ring-C-seco limonoids, and the detailed chemical information will be discussed in this review (Table 1).

**TABLE 1:** Classifications and sources of limonoids isolated from genus *Melia*.

No	Compounds	Part of plant	Sources	Reference
	***Trichilin class***			
1	Azedarachin A	Stem barks	*M. toosendan*	Zhou *et al*. (1996)
2	12-*O-*Acetylazedarachin A	Root barks	*M. toosendan*	Nakatani (1999)
3	12-*O*-Acetylazedarachin B	Stem barks	*M. toosendan*	Zhou *et al*. (1996)
4	Azedarachin C	Root barks	*M. azedarach*	D'Ambrosio and Guerriero (2002)
5	Azedarachin B	Root barks	*M. toosendan*	Nakatani (1999)
6	Sendanin	Stem and root barks	*M. toosendan*	Nakatani (1999)
7	12-Hydroxyamoorastatin	Root barks	*M. toosendan*	Nakatani (1999)
8	Toosendanin	Stem barks	*M. toosendan*	Zhang *et al*. (2010c)
9	12-*O*-Deacetyltrichilin H	Fruits	*M. azedarach*	Zhou *et al*. (2005)
10	12-Acetyltrichilin B	Root barks	*M. azedarach*	Nakatani *et al*. (1994)
11	7,12-Diacetyltrichilin B	Root barks	*M. azedarach*	Nakatani *et al*. (1994)
12	Trichilin H	Stem barks	*M. toosendan*	Zhou *et al*. (1996)
13	Trichilin B	Root barks	*M. toosendan*	Nakatani (1999)
14	Trichilin D	Root barks	*M. azedarach*	Nakatani *et al*. (1994)
15	Meliatoxin A2	Root barks	*M. azedarach*	Nakatani *et al*. (1994)
16	Trichilin K	Stem barks	*M. toosendan*	Zhou *et al*. (1996)
17	Trichilin L	Stem barks	*M. toosendan*	Zhou *et al*. (1996)
18	Trichilin I	Stem barks	*M. toosendan*	Zhou *et al*. (1996)
19	Trichilin J	Stem barks	*M. toosendan*	Zhou *et al*. (1996)
20	12-Deacetyltrichilin I	Barks	*M. azedarach*	Takeya *et al*. (1996b)
21	1-Acetyltrichilin H	Barks	*M. azedarach*	Takeya *et al*. (1996b)
22	3-Deacetyltrichilin H	Barks	*M. azedarach*	Takeya *et al*. (1996b)
23	1-Acetyl-3-deacetyltrichilin H	Barks	*M. azedarach*	Takeya *et al*. (1996b)
24	1-Acetyl-2-deacetyltrichilin H	Barks	*M. azedarach*	Takeya *et al*. (1996b)
25	1,12-Diacetyltrichilin B	Barks	*M. azedarach*	Takeya *et al*. (1996b)
26	Meliatoosenin C	Stem barks	*M. toosendan*	Zhang *et al*. (2010c)
27	24-Norchola-20,22-diene-4-carboralde-hyde-14,15:21,23-diepoxy-1,3,7,12,19-pentachydroxy-4,8-dimethyl-11-oxo-cyclic-4,19-hemiacetal[C(S),1α,3α,4β,5α,7α,12α,13α,14β,15β,17α]-(9Cl)	Stem barks	*M. toosendan*	Zhang *et al*. (2010c)
28	Meliatoosenin D	Stem barks	*M. toosendan*	Zhang *et al*. (2010c)
29	1-*O*-Acetyltrichilin H	Root barks	*M. toosendan*	Zhou *et al*. (1998)
30	12-Acetoxyamoorastatin	Barks	*M. azedarach*	Ahn *et al*. (1994)
31	7-Benzoyltoosendanin	Seeds	*M. azedarach*	Liu *et al*. (2011)
32	7-Cinnamoyltoosendanin	Seeds	*M. azedarach*	Liu *et al*. (2011)
33	Meliarachin B	Twigs and leaves	*M. azedarach*	Su *et al*. (2011)
34	Meliarachin C	Twigs and leaves	*M. azedarach*	Su *et al*. (2011)
35	Meliatoxin B1	Fruits	*M. toosendan*	Tada *et al*. (1999)
36	12α-Hydroxymeliatoxin B1	Fruits	*M. toosendan*	Zhu *et al*. (2014)
37	12α-Acetoxylmeliatoxin B2	Fruits	*M. toosendan*	Zhu *et al*. (2014)
38	Meliatoosenin G	Fruits	*M. toosendan*	Zhang *et al*. (2012)
39	Meliatoosenin H	Fruits	*M. toosendan*	Zhang *et al*. (2012)
40	29-[(2-Methylbutanoyl)oxy]-2*α*-hydroxyamoorastatone	Root barks	*M. toosendan*	Zhou *et al*. (2009)
41	1,3-epi-29-[(2-Methylpropanoyl)oxy]-2*α*-hydroxyamoorastatone	Root barks	*M. toosendan*	Zhou *et al*. (2009)
42	Isotoosendanin	Fruits	*M. toosendan*	Xie *et al*. (2008)
43	Meliarachin L	Fruits	*M. toosendan*	Yan *et al*. (2020)
44	Neoazedarachin A	Root barks	*M. toosendan*	Zhou *et al*. (1998)
45	Neoazedarachin B	Root barks	*M. toosendan*	Zhou *et al*. (1998)
46	Neoazedarachin D	Root barks	*M. toosendan*	Zhou *et al*. (1998)
47	12*α*-Hydroxyamoorastatone	Root barks	*M. toosendan*	Zhou *et al*. (1998)
48	Amoorastatone	Fruits	*M. toosendan*	Wang *et al*. (2020a)
49	12-Dehydroneoazedarachin D	Fruits	*M. azedarach*	Akihisa *et al*. (2013)
50	Meliatoxin B2	Barks	*M. azedarach*	Huang *et al*. (1994)
51	Meliarachin G	Twigs and leaves	*M. azedarach*	Su *et al*. (2011)
52	Meliarachin H	Twigs and leaves	*M. azedarach*	Su *et al*. (2011)
53	Meliarachin I	Twigs and leaves	*M. azedarach*	Su *et al*. (2011)
54	Meliarachin J	Twigs and leaves	*M. azedarach*	Su *et al*. (2011)
55	Meliarachin K	Twigs and leaves	*M. azedarach*	Su *et al*. (2011)
56	12*α*-Hydroxymeliatoosenin I	Fruits	*M. toosendan*	Zhu *et al*. (2014)
57	Meliatoosenin I	Fruits	*M. toosendan*	Zhang *et al*. (2012)
58	Meliatoosenin J	Fruits	*M. toosendan*	Zhang *et al*. (2012)
59	Mesendanin H	Twigs and leaves	*M. toosendan*	Dong *et al*. (2010)
60	Meliarachin D	Twigs and leaves	*M. azedarach*	Su *et al*. (2011)
61	Meliarachin E	Twigs and leaves	*M. azedarach*	Su *et al*. (2011)
62	Meliazedalides B	Fruits	*M. azedarach*	Qiu *et al*. (2019)
63	Toosendalactonins	Fruits	*M. azedarach*	Park *et al*. (2020)
64	Meliatoosenin E	Fruits	*M. toosendan*	Zhang *et al*. (2012)
65	Mesendanin G	Twigs and leaves	*M. toosendan*	Dong *et al*. (2010)
66	Meliartenin	Fruits	*M. azedarach*	Carpinella *et al*. (2003)
67	Toosendanal	Fruits	*M. toosendan*	Tada *et al*. (1999)
	***Vilasinin class***			
68	Meliatoosenin K	Fruits	*M. toosendan*	Zhang *et al*. (2012)
69	Trichilinin D	Root barks	*M. toosendan*	Nakatani (1999)
70	Trichilinin E	Root barks	*M. toosendan*	Nakatani (1999)
71	Toosendansin H	Fruits	*M. toosendan*	Li *et al*. (2020b)
72	1-*O*-cinnamoyltrichilinin	Fruits	*M. toosendan*	Nakatani *et al*. (2000)
73	Trichilinin B	Root barks	*M. toosendan*	Nakatani (1999)
74	Trichilinin C	Root barks	*M. toosendan*	Nakatani (1999)
75	24, 25,26, 27-Tetranorapotirucalla-(apoeupha)-1*α*-tigloyloxy-3*α*, 7*α*-dihydroxyl-12*α*-acetoxyl-14, 20, 22-trien-21, 23-epoxy-6,28-epoxy	Fruits	*M. toosendan*	Zhang *et al*. (2010d)
76	Meliavolkin	Barks	*M. volkensii*	Zeng *et al*. (1995a)
77	Meliavolkinin	Barks	*M. volkensii*	Rogers *et al*. (1998a)
78	1,3-Diacetylvilasinin	Barks	*M. volkensii*	Rogers *et al*. (1998a)
79	1-Acetyltrichilinin	Fruits	*M. volkensii*	Jaoko *et al*. (2020)
80	1-Tigloyltrichilinin	Fruits	*M. volkensii*	Jaoko *et al*. (2020)
81	11,15-Dioxotrichilinin	Fruits	*M. toosendan*	Zhu *et al*. (2014)
82	Toosendansin I	Fruits	*M. toosendan*	Li *et al*. (2020b)
	***Havanensin class***			
83	Mesendanin C	Twigs and leaves	*M. toosendan*	Dong *et al*. (2010)
84	Mesendanin D	Twigs and leaves	*M. toosendan*	Dong *et al*. (2010)
85	Sendanal B	Fruits	*M. toosendan*	Yan *et al*. (2020)
86	24,25,26,27-Tetra-norapotirucalla-(apoeupha)-1*α*,6*α*,12*α*-triacetoxyl-3*α*,7*α*-dihydroxyl-28-aldehyde-14,20,22-trien-21,23-epoxy	Fruits	*M. toosendan*	Zhang *et al*. (2013)
87	14,15-Deoxy-11-oxohavanensin 3,12-diacetate	Fruits	*M. toosendan*	Zhu *et al*. (2014)
88	Mesendanin A	Twigs and leaves	*M. toosendan*	Dong *et al*. (2010)
89	Mesendanin B	Twigs and leaves	*M. toosendan*	Dong *et al*. (2010)
90	Mesendanin J	Twigs and leaves	*M. toosendan*	Dong *et al*. (2010)
91	Toosendone	Fruits	*M. toosendan*	Zhang *et al*. (2007)
92	Butenolide	Root barks	*M. toosendan*	Nakatani (1999)
93	Meliarachin A	Twigs and leaves	*M. azedarach*	Su *et al*. (2011)
94	Melianin C	Barks	*M. volkensii*	Rogers *et al*. (1998a)
95	Meliatoosenin F	Fruits	*M. toosendan*	Zhang *et al*. (2012)
96	Mesendanin I	Twigs and leaves	*M. toosendan*	Dong *et al*. (2010)
	***Azadirone class***			
97	Meliatoosenin A	Stem barks	*M. toosendan*	Zhang *et al*. (2010c)
98	Azadirone	Root barks	*M. toosendan*	Nakatani (1999)
99	Acetyltrichilenone	Root barks	*M. toosendan*	Nakatani (1999)
	***Nimbolinin class***			
100	12-*O*-Methylvolkensin	Fruits	*M. toosendan*	Tada *et al*. (1999)
101	1-Deacetylnimbolinin A	Root barks	*M. toosendan*	Nakatani *et al*. (1999)
102	Nimbolinin A	Fruits	*M. toosendan*	Su *et al*. (2013)
103	Nimbolinin C	Fruits	*M. toosendan*	Nakatani *et al*. (2000)
104	Nimbolinin D	Fruits	*M. toosendan*	Nakatani *et al*. (2000)
105	Nimbolinin B	Fruits	*M. toosendan*	Su *et al*. (2013)
106	1-Deacetylnimbolinin B	Fruits	*M. toosendan*	Su *et al*. (2013)
107	12-Ethoxynimbolinins A	Fruits	*M. toosendan*	Zhang *et al*. (2007)
108	12-Ethoxynimbolinins B	Fruits	*M. toosendan*	Zhang *et al*. (2007)
109	12-Ethoxynimbolinins C	Fruits	*M. toosendan*	Zhang *et al*. (2007)
110	12-Ethoxynimbolinins D	Fruits	*M. toosendan*	Zhang *et al*. (2007)
111	12-Ethoxynimbolinins H	Barks	*M. toosendan*	Zhang *et al*. (2018)
112	Nimbolin B	Root barks	*M. volkensii*	Zeng *et al*. (1995b)
113	1-Decinnamoyl-1-(2′-methylacryloyl) nimbolinin C	Fruits	*M. toosendan*	Zhu *et al*. (2014)
114	1-Decinnamoylnimbolinin C	Fruits	*M. toosendan*	Zhu *et al*. (2014)
115	12-*O*-Methyl-1-*O*-deacetylnimbolinin B	Fruits	*M. toosendan*	Hu *et al*. (2011)
116	12-*O*-Methyl-1-*O*-tigloyl-1-*O*-deacetylnimbolinin B	Fruits	*M. toosendan*	Hu *et al*. (2011)
117	12-*O*-Ethylnimbolinin B	Fruits	*M. toosendan*	Hu *et al*. (2011)
118	1*α*, 7*α*-Dihydroxyl-3*α*-acetoxyl-12*α*-ethoxylnimbolinin	Fruits	*M. toosendan*	Zhang *et al*. (2016)
119	1*α*-Tigloyloxy-3*α*-acetoxyl-7*α*-hydroxyl-12*β*-ethoxylnimbolinin	Fruits	*M. toosendan*	Zhang *et al*. (2016)
120	1*α*, 3*α*-Dihydroxyl-7*α*-tigloyloxy-12*α*-ethoxylnimbolinin	Fruits	*M. toosendan*	Zhang *et al*. (2016)
121	1*α*, 7*α*-Ditigloyloxy-3*α*-acetoxyl-12*α*-ethoxylnimbolinin	Fruits	*M. toosendan*	Zhang *et al*. (2016)
122	1*α*-Benzoyloxy-3*α*-acetoxyl-7*α*-hydroxyl-12*β*-ethoxylnimbolinin	Fruits	*M. toosendan*	Zhang *et al*. (2013)
123	12*α*-1-*O*-Tigloyl-1-*O*-deacetyl-nimbolinin B	Fruits	*M. toosendan*	Su *et al*. (2013)
124	1*α*-Benzoyloxy-3*α*-acetoxyl-7*α*-hydroxyl-12*α*-ethoxyl nimbolinin	Fruits	*M. toosendan*	Zhang *et al*. (2010b)
125	1-*O*-Benzoyl-3-*O*-deactylnim-bolinin C	Fruits	*M. azedarach*	Park *et al*. (2020)
126	1-Benzoylnimbolinin C	Seeds	*M. azedarach*	Liu *et al*. (2011)
127	Meliatoosenin L	Fruits	*M. toosendan*	Zhang *et al*. (2012)
128	Meliatoosenin N	Fruits	*M. toosendan*	Zhang *et al*. (2012)
129	Toosendansins B	Fruits	*M. toosendan*	Chen *et al*. (2014)
130	3-Deacetyl-12-*O*-methylvolkensin	Fruits	*M. toosendan*	Zhu *et al*. (2014)
131	Meliatoosenin T	Fruits	*M. toosendan*	Yan *et al*. (2020)
132	Meliatoosenin U	Fruits	*M. toosendan*	Yan *et al*. (2020)
133	Meliazedalides A	Fruits	*M. azedarach*	Qiu *et al*. (2019)
	***Ohchinolide class***			
134	Ohchinolide A	Fruits	*M. azedarach*	Ochi *et al*. (1979)
135	Ohchinolide B	Root barks	*M. toosendan*	Zhou *et al*. (1997)
136	Ohchinolide C	Barks	*M. toosendan*	Zhou *et al*. (1997)
137	1-*O*-Deacetyl-1-*O*-tigloylohchinolide B	Fruits	*M. azedarach*	Zhou *et al*. (2004)
138	1-*O*-Deacetyl-1-*O*-benzoylohchinolide B	Fruits	*M. azedarach*	Zhou *et al*. (2004)
139	1-*O*-Deacetyl-1-*O*-tigloylohchinolide A	Fruits	*M. azedarach*	Zhou *et al*. (2004)
140	1-*O*-Deacetylohchinolide B	Fruits	*M. azedarach*	Zhou *et al*. (2004)
141	1-*O*-Deacetylohchinolide A	Fruits	*M. azedarach*	Zhou *et al*. (2004)
142	Azecin 2	Roots	*M. azedarach*	Tan and Luo (2011)
	***Nimbolidin class***			
143	Nimbolidin F	Barks	*M. toosendan*	Zhou *et al*. (1997)
144	Nimbolidin D	Barks	*M. toosendan*	Zhou *et al*. (1997)
145	Nimbolidin C	Root barks	*M. toosendan*	Nakatani *et al*. (1996)
146	Nimbolidin E	Root barks	*M. toosendan*	Nakatani *et al*. (1996)
147	Nimbolidin B	Root barks	*M. toosendan*	Nakatani *et al*. (1996)
148	15-*O*-Deacetyl-15-*O*-methylnimbolidin A	Fruits	*M. azedarach*	Zhou *et al*. (2005)
149	15-*O*-Deacetyl-15-*O*-methylnimbolidin B	Fruits	*M. azedarach*	Zhou *et al*. (2005)
150	15-*O*-Deacetylnimbolidin B	Fruits	*M. azedarach*	Zhou *et al*. (2005)
	***Salannin class***			
151	1-*O*-Cinnamoyl-1-*O*-debenzoylohchinal	Fruits	*M. toosendan*	Hu *et al*. (2011)
152	1-*O*-Tigloyl-1-*O*-debenzoylohchinal	Fruits	*M. toosendan*	Hu *et al*. (2011)
153	Salannin	Root barks	*M. toosendan*	Nakatani *et al*. (1996)
154	3-*O*-Deacetylsalanni	Fruits	*M. azedarach*	Akihisa *et al*. (2013)
155	Ohchinal	Fruits	*M. toosendan*	Wang *et al*. (2020a)
156	Ohchinin	Leaves	*M. azedarach*	Pan *et al*. (2014b)
157	1-*O*-Decinnamoyl-1-*O*-benzoylohchinin	Fruits	*M. azedarach*	Akihisa *et al*. (2013)
158	3-Deacetyl-4′-demethylsalannin	Fruits	*M. azedarach*	Pan *et al*. (2014a)
159	3-Deacetyl-3-tigloylsalannin	Fruits	*M. azedarach*	Akihisa *et al*. (2017)
160	2′,3′-Dihydrosalannin	Fruits	*M. volkensii*	Jaoko *et al*. (2020)
161	1-Detigloyl-1-isobutylsalannin	Fruits	*M. volkensii*	Jaoko *et al*. (2020)
162	Ohchinin-acetate	Fruits	*M. volkensii*	Jaoko *et al*. (2020)
163	Ohchininolide	Fruits	*M. azedarach*	Akihisa *et al*. (2013)
164	1-*O*-Decinnamoyl-1-*O*-benzoylohchininolide	Fruits	*M. azedarach*	Akihisa *et al*. (2013)
165	23-Hydroxyohchininolide	Leaves	*M. azedarach*	Pan *et al*. (2014b)
166	23-Methoxyohchininolide A	Fruits	*M. azedarach*	Akihisa *et al*. (2013)
167	1-*O*-Decinnamoyl-1-*O*-benzoyl-23-hydroxyohchininolide	Fruits	*M. azedarach*	Akihisa *et al*. (2013)
168	3-Deacetyl-28-oxosalannin	Leaves	*M. azedarach*	Pan *et al*. (2014b)
169	3-Deacetyl-4′-demethyl-28-oxosalannin	Leaves	*M. azedarach*	Pan *et al*. (2014b)
170	1-*O*-Decinnamoyl-1-*O*-benzoyl-28-oxoohchinin	Fruits	*M. azedarach*	Akihisa *et al*. (2013)
171	3-Deacetyl-28-oxosalannolactone	Leaves	*M. azedarach*	Pan *et al*. (2014b)
172	3-Deacetyl-28-oxoisosalanninolide	Leaves	*M. azedarach*	Pan *et al*. (2014b)
173	3-Deacetyl-17-defurano-17,28-dioxosalannin	Leaves	*M. azedarach*	Pan *et al*. (2014b)
174	21-Hydroxyisoohchininolide	Leaves	*M. azedarach*	Akihisa *et al*. (2013)
175	17-Defurano-17-oxoohchinin	Fruits	*M. azedarach*	Akihisa *et al*. (2013)
176	Meliatoosenin P	Fruits	*M. toosendan*	Zhang *et al*. (2012)
177	Meliatoosenin Q	Fruits	*M. toosendan*	Zhang *et al*. (2012)
	***Ohchinolal class***			
178	3-*O*-Acetylohchinolal	Barks	*M. toosendan*	Zhou *et al*. (1997)
179	1-Detigloylohchinolal	Fruits	*M. azedarach*	Pan *et al*. (2014a)
180	1-Benzoyl-1-detigloylohchinolal	Fruits	*M. azedarach*	Akihisa *et al*. (2017)
181	1-Cinnamoyl-1-detigloylohchinolal	Fruits	*M. azedarach*	Akihisa *et al*. (2017)
182	Ohchinolal	Root barks	*M. toosendan*	Zhou *et al*. (1997)
183	Mesendanin E	Fruits	*M. azedarach*	(Dong *et al*., 2010; Pan *et al*., 2014a)
184	Mesendanin F	Twigs and leaves	*M. toosendan*	Dong *et al*. (2010)
185	Toosendansin G	Fruits	*M. toosendan*	Li *et al*. (2020b)
186	Meliatoosenin R	Fruits	*M. toosendan*	Zhang *et al*. (2012)
	***Meliacarpinin class***			
187	Toosendane A	Barks	*M. toosendan*	Hu *et al*. (2018)
188	Toosendane B	Barks	*M. toosendan*	Hu *et al*. (2018)
189	Toosendane C	Barks	*M. toosendan*	Hu *et al*. (2018)
190	3,20-Diacetyl-11-methoxymeliacarpinin	Barks	*M. toosendan*	Hu *et al*. (2018)
191	3,20-Diacetyl-1-tigloyl-11-methoxymeliacarpinin	Barks	*M. toosendan*	Hu *et al*. (2018)
192	Meliacarpinin A	Root barks	*M. toosendan*	Nakatani (1999)
193	Meliacarpinin C	Root barks	*M. toosendan*	Nakatani (1999)
194	Meliacarpinin D	Root barks	*M. toosendan*	Nakatani (1999)
195	Meliacarpinin E	Root barks	*M. azedarach*	Huang *et al*. (1996)
196	1-Deoxy-3-tigloyl-11-methoxymeliacarpinin	Barks	*M. azedarach*	Huang *et al*. (1994)
197	3*α*-(2-Methylbutyryl)-1,20-diacetyl-11-methoxymeliacarpinin	Twigs and leaves	*M. azedarach*	Zhang *et al*. (2014)
198	3-Tigloyl-1,20-diacetyl-11-methoxymeliacarpinin	Twigs and leaves	*M. azedarach*	Zhang *et al*. (2014)
199	1-Methacrylyl-3-acetyl-11-methoxymeliacarpinin	Roots	*M. azedarach*	Nakatani (1999)
200	1-(2-Methylpropanoyl)-3-acetyl-11-methoxymeliacarpinin	Roots	*M. azedarach*	Nakatani (1999)
201	1-Cinnamoyl-3-hydroxy- 11-methoxymeliacarpinin	Barks	*M. azedarach*	Takeya *et al*. (1996a)
202	1-Deoxy-3-methacrylyl-11-methoxymeliacarpinin	Barks	*M. azedarach*	Takeya *et al*. (1996a)
	***Meliacarpin class***			
203	1,3-Dicinnamoyl-11-hydroxymeliacarpin	Leaves	*M. azedarach*	Bohnenstengel *et al*. (1999)
204	1-Cinnamoyl-3-methacrylyl-11-hydroxymeliacarpin	Leaves	*M. azedarach*	Bohnenstengel *et al*. (1999)
205	1-Cinnamoyl-3-acetyl-11-hydroxymeliacarpin	Leaves	*M. azedarach*	Bohnenstengel *et al*. (1999)
206	1-Cinnamoyl-3-feruloyl-11-hydroxymeliacarpin	Leaves	*M. azedarach*	Bohnenstengel *et al*. (1999)
207	Azadirachtin	Leaves	*M. azedarach*	Bohnenstengel *et al*. (1999)
208	1-Cinnamoyl-3,11-dihydroxymeliacarpin	Leaves	*M. azedarach*	Barquero *et al*. (2006)
	***Others***			
209	Meliatoosenin S	Fruits	*M. toosendan*	Zhang *et al*. (2012)
210	Spirosendan	Root barks	*M. toosendan*	Nakatani *et al*. (1999)
211	Volkensinin	Barks	*M. volkensii*	Rogers *et al*. (1998b)
212	Azecin 1	Roots	*M. azedarach*	Tan and Luo (2011)
213	Azecin 3	Roots	*M. azedarach*	Tan and Luo (2011)
214	Azecin 4	Roots	*M. azedarach*	Tan and Luo (2011)

### Trichilin Class (1–67)

**FIGURE 2:**
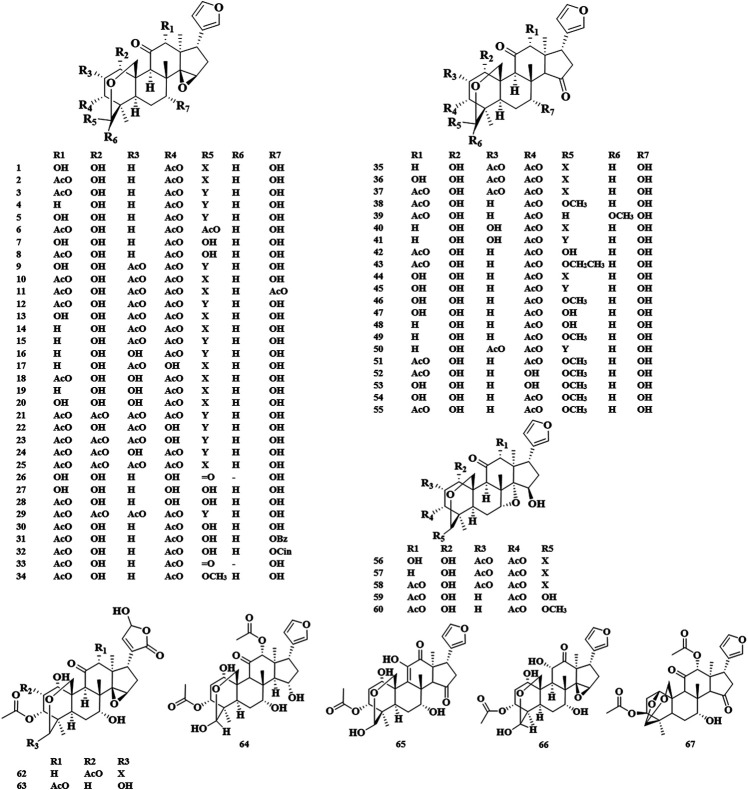
Structures of trichilin class limonoids 1–67.

The basic skeleton of the trichilin class limonoids contained the C-19/29 bridged acetal and the 14,15-epoxide moieties or C15 carbonyl. Toosendanin, as a characteristic compound in trichilin class, has been confirmed to be a potential bioactive component in the field of anti-tumor, insecticide, and anti-botulism. It should be noted that toosendanin can only be isolated from two plant species, *M. toosendan* and *M. azedarach* (Shi and Li, 2007). The first isolation and identification of toosendanin can be traced back to 1975. In 1980, Chinese researchers corrected its chemical structure due to the occurrence of two tautomers found by paper chromatography and silica gel plate method (Su and Liang, 1980). In 1984, iso-toosendanin, the isomer of toosendanin, was also isolated from these two plants. In 1998, Zhou *et al.* isolated four new trichilin class limonoids from the root barks of *M. toosendan*, of which neoazedarachin D was found to be the first natural 29-endo-derivative in C (19)/C (29) bridged acetal limonoids (Zhou et al., 1998). Apart from C-19/29 bridged acetal, toosendanal with C (1)/C (29) bridged acetal was also discovered in *M. toosendan* (Tada et al., 1999) (Figure 2).

### Vilasinin Class (68–82)

**FIGURE 3:**
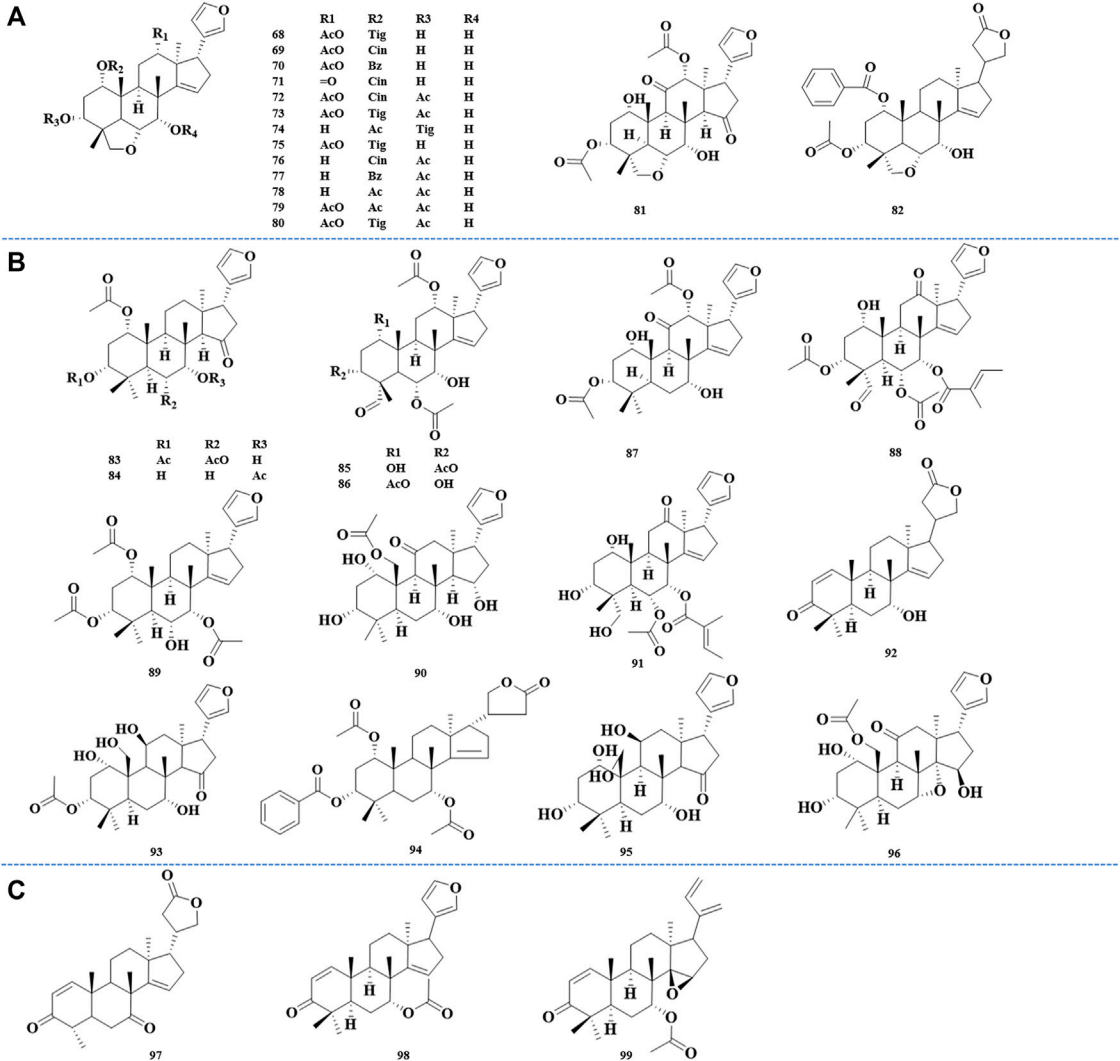
Structures of vilasinin class limonoids 68–82 **(A)**, structures of havanensin class limonoids 83–96 **(B)**, and structures of azadirone class limonoids 97––99 **(C)**.

6*α*,28-ether bridge was supposed to be a typical skeleton of vilasinin class limonoids. Trichilinin B, C, D, and E, four new limonoids with 6*α*,28-ether bridge, were isolated and identified from the root barks of *M. toosendan*. It was found that the unique C-12 oxygen function in trichilinin B, C, and E seemed to be the biosynthetic precursors of ring-C-cleaved limonoids (Nakatani, 1999). Using activity-guided separation, meliavolkin was isolated from *M. volkensii* Gürke., and its structure was elucidated by MS, ^1^H and ^13^C NMR, COSY, NOESY, HETCOR, and COLOC spectra (Zeng et al., 1995a) (Figure 3A).

### Havanensin Class (83–96)

The havanensin class limonoids were characteristic of C-1, C-3, and C-7 oxygen substituents, and C-28 will be oxidized, which varies from methyl to carboxyl. In 1998, melianin C was isolated from *M. volkensii* Gürke., which was confirmed to have a consistent structure with the semisynthetic compound obtained by Jones oxidation of melianin A, and it was also the first report that melianin C appeared naturally (Rogers LL. et al., 1998). Sendanal was associated closely with a precursor of the 14,15-epoxy-12-hydroxymoiety, which could produce the ring-seco limonoids through a Grob fragmentation followed by the formation of an ether ring between the C-7 and C-15 hydroxyl groups *via* an S_N_1 mechanism (Tan and Luo, 2011) (Figure 3B).

### Azadirone Class (97–99)

The 3-oxo-Δ^1,2^ and C-7 oxygenation was considered as a vital feature of azadirone-type limonoids. The first isolation of azadirone can be traced back to 1998, and it was firstly isolated from the root barks of *M. toosendan*, together with acetyltrichilenone (Nakatani, 1999) (Figure 3C).

### Ring-Seco Limonoids (100–208)

**FIGURE 4:**
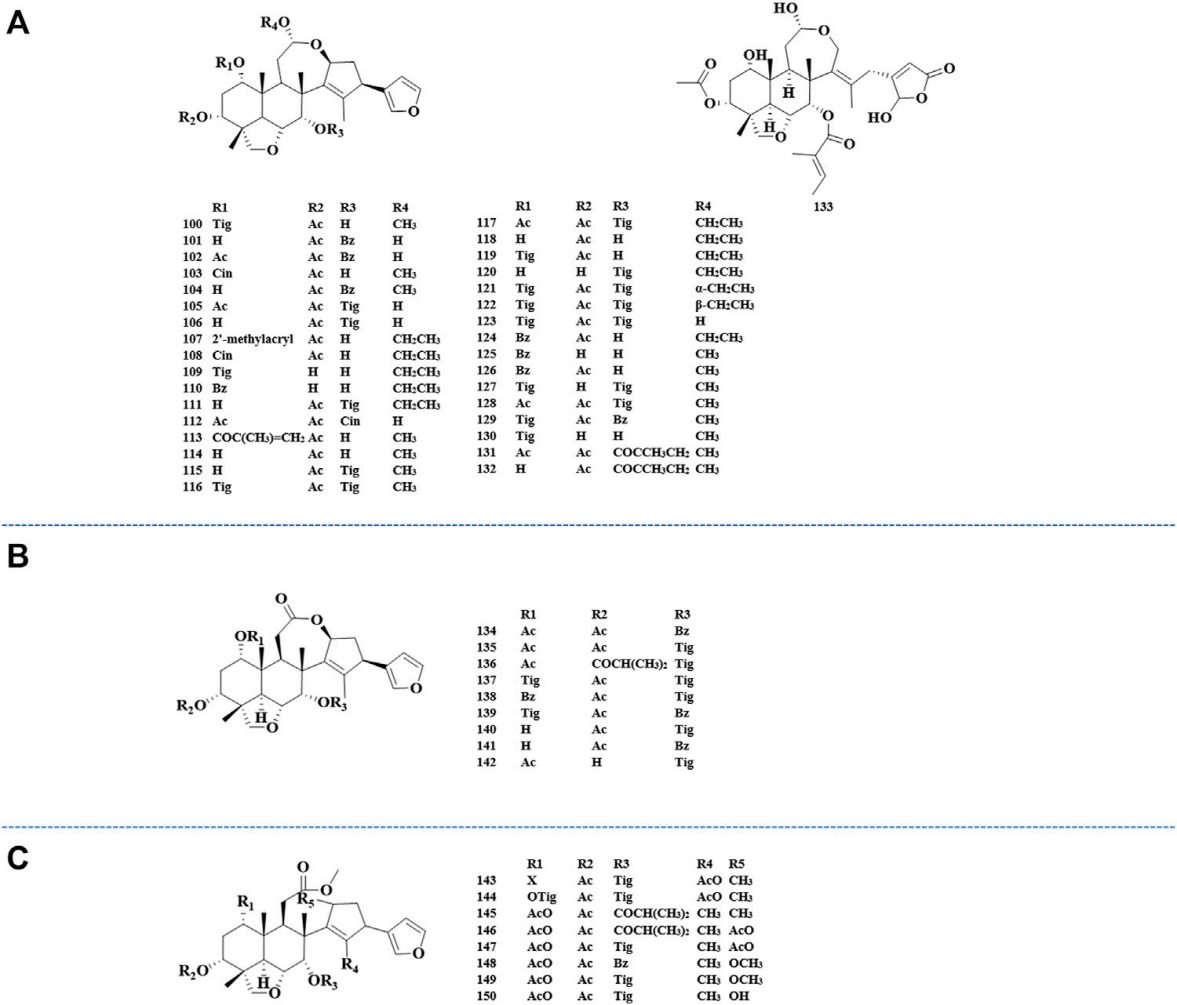
Structures of nimbolinin class limonoids 100–133 **(A)**, structures of ohchinolide class limonoids 134–142 **(B)**, and structures of nimbolidin class limonoids 143–150 **(C)**.

**FIGURE 5:**
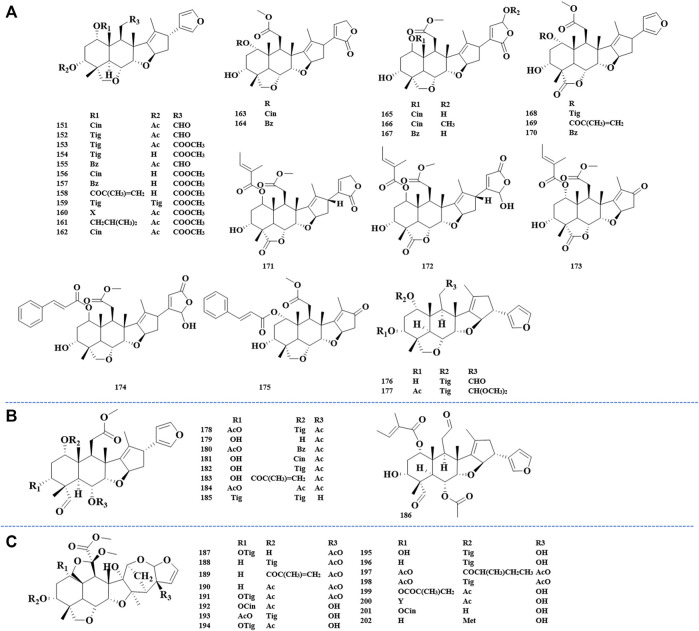
Structures of salannin class limonoids 151–177 **(A)**, structures of ohchinolal class limonoids 178–186 **(B)**, and structures of meliacarpinin class limonoids 187–202 **(C)**.

**FIGURE 6:**
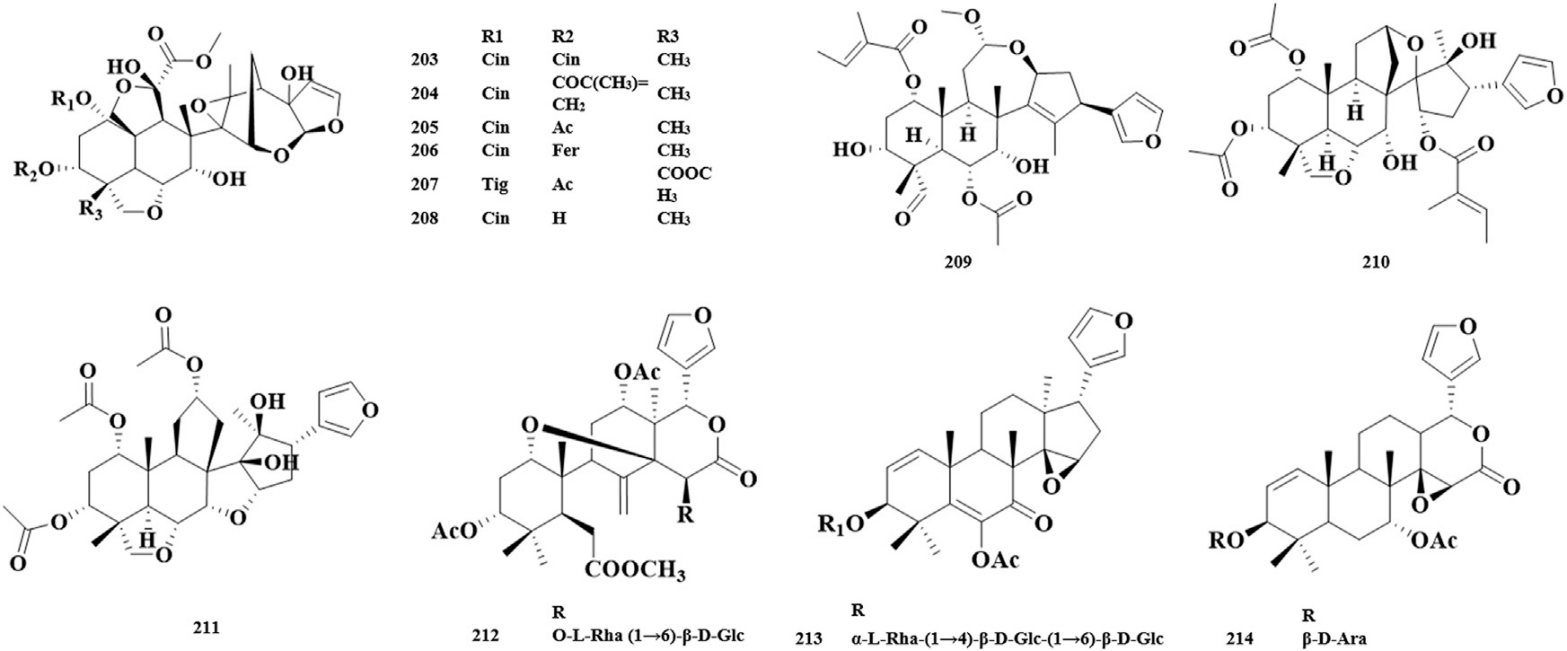
Structures of meliacarpin class limonoids 203–208 **(A)** and structures of other-class limonoids 209–214 **(B)**. Rha, rhamnose; Glc, clucose; Ara, arabinose.

The ring-C-seco limonoids have the most typical chemical structure of all ring-seco limonoids. According to different structural characteristics, the ring C-seco limonoids can be classified into nimbolinin class, ohchinolide class, nimbolidin class, salannin class, ohchinolal class, meliacarpinin class, and meliacarpin class (Figures 4–6).

**FIGURE 7:**
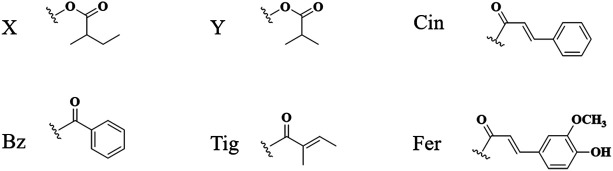
Abbreviation of structure.

## Synthesis of Limonoids

**FIGURE 8:**
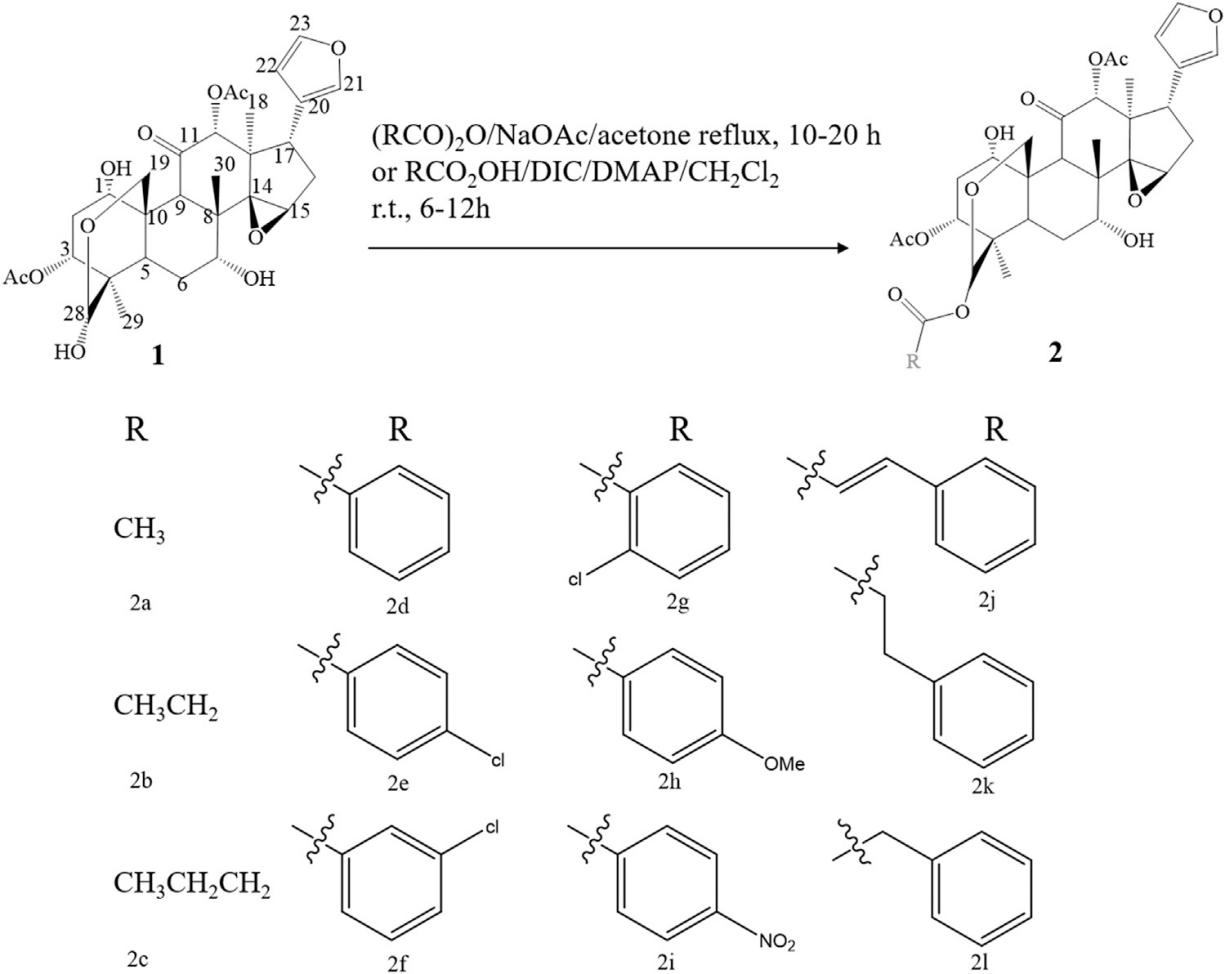
The synthetic route of 28-acyloxy derivatives of toosendanin.

Due to the remarkable pharmacological activities and the unique structural features, many endeavors have been devoted to the synthesis of limonoids found in the genus *Melia*. Inspired by the anti-botulinum effect and structural diversity of toosendanin, researchers have conducted a synthesis or structural modification of this chemical structure. Excitedly, a similar framework can be found in all mammalian steroids and hormones. Although total synthesis seems like an ideal synthesis approach, the complexity of structure and uncertainty of total synthesis make researchers choose function-oriented synthesis (FOS) as an alternative. FOS aims to emulate a bioactive lead structure or possibly substitute for it with simpler scaffolds designed to contain the active chemical structure sites of target compounds (Wender, 2013). Considering that there were no previous reports on bioactive structural features, Nakai *et al.* cleaved toosendanin into AB and CD rings, and the biological activity of CD rings was probed by mouse lethality assay, which was deemed to be a “gold standard” in the validation of anti-botulinum activity. Using mesityl oxide and acetylacetone as synthetic substrates, a 4-acetoxy CD fragment analogue of toosendanin was synthesized in 14 steps. Unluckily, no significant anti-botulinum effect was found in the obtained 4-acetoxy CD fragment. Nevertheless, this synthetic method can provide certain inspiration for synthesizing the CD ring analogues (Nakai et al., 2009). Later, in 2010, Nakai *et al.* focused on AB ring synthesis, which contained a unique bridged hemiacetal, and the anti-botulinum effect was tested in a rat spinal cord cellular assay. The AB ring and epi-AB ring were successfully synthesized with a yield rate of 5.8 and 10% after 23 steps and 19 steps, respectively. Similar to the previously synthesized CD ring, the AB ring did not exhibit anti-botulism activity. All of these results indicated that the complete structure of the ABCD ring is indispensable in the treatment of botulism (Nakai et al., 2010). Apart from exploring the influence of ring structure on the bioactivity of toosendanin, the role of substituents at different positions was investigated as well. By modifying the C-28 position of toosendanin, 12 28-acyloxy derivatives were semi-synthesized, and the insecticidal activity against *Mythimna separata* Walker was evaluated. The outcomes revealed that the butanoyloxy and phenyl acryloyl oxymoiety at the 28-position plays a critical role in insecticidal activity (Xu and Zhang, 2011) (Figure8). Except for the organic synthesis mentioned above, the biosynthesis of toosendanin also aroused great attention. Since the cyclization of 2,3-oxidosqualene by oxidosqualene cyclase (OSC) is the first stage of triterpenoid biosynthesis to produce diverse triterpene frameworks, Lian *et al.* found potential OSC genes in *M. toosendan* by utilizing the triterpenoid profile, transcriptome data, and phylogenetic analysis. The following functional analysis discovered that MtOSC1 was a crucial enzyme in the formation of tirucalla-7,24-dien-3β-ol, which is a precursor for the biosynthesis of toosendanin (Lian et al., 2020). This study revealed that the practicable biosynthesis route of toosendanin may promote its application both in agricultural and medicinal use. Furthermore, salannin and 3-deacetylsalannin can be transformed into 3-deacetoxy-1-de[(E)-2-methylbut-2-enoloxy]salannin-1-en-3-one by *Nocardia* sp., and the transformed product was validated as a candidate bioactive compound with an α,β-unsaturated ketone moiety in ring A (Madyastha and Venkatakrishnan, 2000).

## Biological Activities of Limonoids

### Anti-tumor Effects

Accumulating studies have found that limonoids possessed broad-spectrum anti-tumor activities against multiple tumors, such as gastric tumor (Shao et al., 2020), lung tumor (Luo et al., 2018), colorectal tumor (Wang et al., 2015), glioblastoma (Cao et al., 2016), leukemia (Ju et al., 2012), ovarian tumor (Li et al., 2018b), hepatocellular carcinoma (He et al., 2010), breast tumor (Wang et al., 2018), Ewing’s sarcoma (Gao et al., 2019), neuroma (Tang et al., 2004), lymphoma (Zhang B. et al., 2005), and pancreatic tumor (Pei et al., 2017), which indicated that limonoids could be candidate anticancer drugs. Toosendanin (TSN), as a quality control marker in *M. toosendan* and *M. azedarach*, has been systematically investigated for its anti-tumor effects. Thus, this review primarily focused on summarizing the anti-tumor activity of TSN and its underlying mechanisms, including the inhibition of tumor cell proliferation, induction of apoptosis, suppression of migration, and invasion. Besides these, we also comprehensively reviewed the anti-tumor bioactivities of other limonoids in genus *Melia* plants.

#### Effect of Toosendanin on Tumor Cell Proliferation and Cell Cycle Arrest

Tumor cell proliferation is the basis of tumor growth and metastasis. Inhibition of tumor cell proliferation has been considered as one of the pivotal anti-tumor therapies (Whitfield et al., 2006). It was found that TSN could inhibit the proliferation of MKN-45 gastric cancer cells in a time-dependent and dose-dependent manner, and the IC_50_ value was 81.06 nM for 48 h (Shao et al., 2020). In addition, some other experiments showed that TSN was able to restrain the proliferation of various cancer cell lines, including SGC-7901 cells (Wang et al., 2017), MGC-803 cells, and HGC-27 cells with IC_50_ values of 0.11 μM (72 h), 20.30 nM (72 h), and 0.56 µM (48 h), respectively. Using the MTT method, Liu *et al.* reported that the proliferation of lung cancer A549 cells could be inhibited by TSN with an IC_50_ value of 40.206 μM for 48 h. Meanwhile, the same inhibitory effect was found in H1975 lung cancer cells. When treating colorectal cancer SW480 cells with TSN, it exhibited anti-proliferation effects with an IC_50_ value of 0.1059 µM (48 h) (Wang et al., 2015). In order to further explore the anti-tumor effect of TSN in colorectal cancer, a nude mouse xenograft model was established, which subcutaneously inoculated HCT116 cells into nude mice. The outcomes indicated that TSN significantly reduced the tumor volume and weight of the HCT116 xenografts (Wang et al., 2020b). Apart from cancer mentioned above, TSN was found to possess anti-proliferation effects on some other types of tumor, such as glioblastoma, leukemia, ovarian cancer, hepatocellular carcinoma, *etc.*, with IC_50_ values ranging from 5.4 to 900 nM (Zhang B. et al., 2005; He et al., 2010; Cao et al., 2016; Gao et al., 2019).

The proliferation of tumor cells needs to go through four different stages: pre-DNA synthesis (G1), DNA synthesis (S), late DNA synthesis (G2), and mitosis (M). Since the aberrant activity of various cyclins can lead to the uncontrolled proliferation of tumor cells, treating aberrant cyclins was thus considered as an attractive target for tumor therapy (Otto and Sicinski, 2017). Shao *et al.* found that TSN-treated MKN-45 human gastric cancer cells markedly arrested at the G1 phase, which was regulated by the overexpression of mir-23a-3p (Shao et al., 2020). In another study, researchers showed that treatment with TSN at concentrations of 2 and 5 μM can cause G1/S arrest in AGS and HGC-27 gastric cancer cells. For a further evaluation of cell cycle arrest, the expression of G1 checkpoint-associated proteins, including cyclin D1 and p21, was assessed. It was observed that TSN treatment could decrease the expression of cyclin D1, while it could increase the expression of p21 (Zhou et al., 2018). Furthermore, in both 5-FU-sensitive and 5-FU-resistant colorectal cancer cells, G1 phase arrest could be induced by TSN. The results also found that, apart from decreased cyclin D1 and increased p21, the expression of p53, a G1-phase checkpoint-associated protein, increased as well (Wang et al., 2020b). In addition, cell cycle arrest also occurred in other tumor cells, such as glioblastoma (Wang Q. et al., 2020) and leukemia (Ju et al., 2013).

#### Effect of Toosendanin on Tumor Cell Apoptosis

**FIGURE 9:**
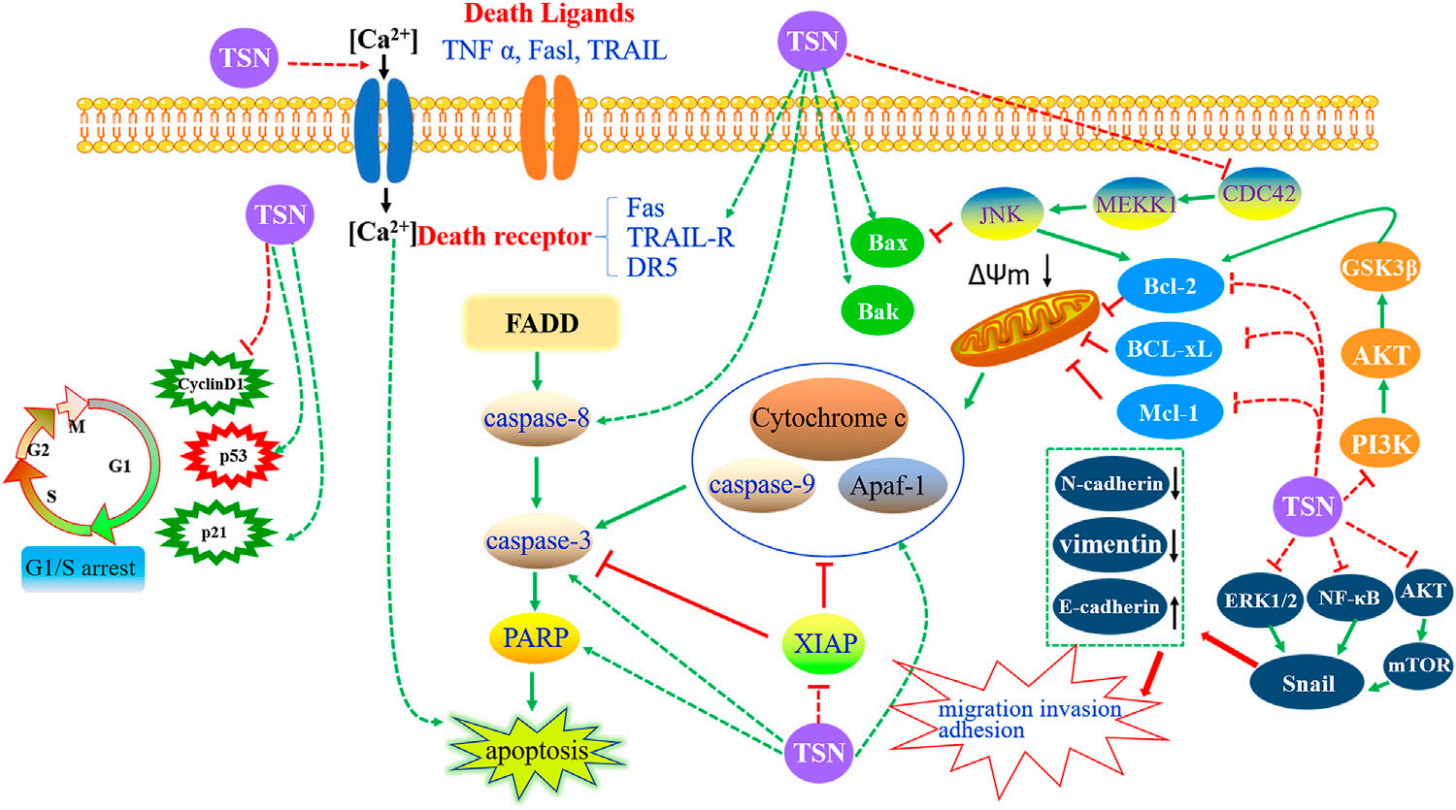
Targeting the apoptosis signaling pathways by toosendanin in cancers.

Concerning the anti-tumor effects of TSN, much work has focused on cell apoptosis. Inducing tumor cell apoptosis is now generally considered as the most important mechanism of anti-tumor drugs. Apoptosis, also known as programmed cell death, is a kind of cell-autonomous and orderly death mode, which is activated, expressed, and regulated by specific genes in cells (Li-Weber, 2013). TSN was recently regarded as a potential candidate that killed tumor cells through the induction of apoptosis in various cancer cells, including gastric cancer (Wang et al., 2017; Zhou et al., 2018; Shao et al., 2020), lung cancer (Wang et al., 2017), colorectal cancer (Wang et al., 2015; Wang et al., 2020b), glioblastoma (Cao et al., 2016; Wang Q. et al., 2020), leukemia (Ju et al., 2012; Ju et al., 2013), ovarian cancer (Li et al., 2018a; Li et al., 2019), hepatocellular carcinoma (He et al., 2010; Liu et al., 2016), breast cancer (Wang et al., 2018), Ewing’s sarcoma (Gao et al., 2019), neuroma (Tang et al., 2004), and lymphoma (Zhang B. et al., 2005). Herein our study summed up the detailed mechanisms of apoptosis induced by TSN (Figure 9).

##### Targeting the Intrinsic Pathway

The intrinsic pathway is also known as cell mitochondrial apoptosis. When cells are exposed to various stimulating factors, such as hypoxia, activation of the oncogene, DNA damage, and cytotrophic factor deficiency, the steady state of mitochondrial membrane potential will be destroyed, and the permeability can increase. Subsequently, the pro-apoptotic factors in the mitochondrial membrane are released to the cytoplasm, which later activates the apoptosis pathway of the mitochondria and causes cell death (Estaquier et al., 2012). Cytochrome c (Cyt C), apoptosis-inducing factor, cysteinyl aspartate-specific proteinase (caspase) activator, and apoptotic protease-activating factor-1 (Apaf-1) are supposed to be pivotal pro-apoptotic factors. The apoptosome complex is formed by the combination of released Cyt C with Apaf-1 and caspase-9, which activates caspase-3 and eventually induces apoptosis. Importantly, regulating the B-cell lymphoma-2 (Bcl-2) protein family has been considered as an essential route to play a role in cell mitochondrial apoptosis signal pathway, the mechanism of which is to control the permeability of the mitochondrial membrane to regulate the release of Cyt C (Siddiqui et al., 2015).

Collectively, TSN-induced apoptosis of tumor cells is related to the activation of the intrinsic pathway. On the one hand, the capacity of cells to undergo mitochondrial apoptosis is governed by pro- and anti-apoptotic members of the Bcl-2 protein family. According to the difference of its functional domain, it can be divided into anti-apoptotic Bcl-2 subfamily (*e.g.*, Bcl-2, Bcl-xL, and Mcl-1), pro-apoptotic Bax subfamily (*e.g.*, Bax and Bak), and BH3-only pro-apoptotic family (*e.g.*, Bid, Bim, Bik, and Noxa) (Campbell and Tait, 2018). On the other hand, the caspase family is known as the most well-studied apoptotic executive protein, which has the characteristics of post-aspartic protein hydrolysis. Based on their different roles in apoptosis, it can be classified into two types: the initiator (apical) caspases (*e.g.*, caspase-2, -8, -9, and -10) and effector (executioner) caspases (*e.g.*, caspase-3, -6, and -7) (Ola et al., 2011). The caspase family implements its pro-apoptotic effect mainly through the combination of apoptosis signal and death receptor to induce the activation process of initiator and effector caspase protein and the subsequent inactivation process of apoptosis-inhibitory protein and cause nucleic acid and cell structure cleavage and so on (Fiandalo and Kyprianou, 2012). When administrated with TSN, several typical characteristics of apoptosis occurred, including nuclei condensation, DNA fragmentation, reduction of mitochondrial membrane potential, and the upregulation of cytochrome c in cytosol (Tang et al., 2004; Shao et al., 2020). Subsequently, caspase 9 and caspase 3 were activated (Tang et al., 2004; He et al., 2010; Ju et al., 2013; Zhou et al., 2018; Gao et al., 2019; Wang et al., 2020b; Shao et al., 2020), and TSN promoted poly-ADP-ribose polymerase (a target protein of caspase 3) fragmentation (Tang et al., 2004; Ju et al., 2013; Zhou et al., 2018; Wang et al., 2020b), eventually leading to apoptosis. Some other researches showed that TSN treatment significantly increased the pro-apoptotic protein expression of Bax (He et al., 2010; Ju et al., 2013; Cao et al., 2016; Liu et al., 2016; Zhou et al., 2018; Gao et al., 2019; Shao et al., 2020), Bak (Cao et al., 2016), Bim, and Noxa Apaf-1 (Shao et al., 2020) and decreased the anti-apoptotic protein expression of Bcl-2 (He et al., 2010; Ju et al., 2013; Cao et al., 2016; Liu et al., 2016; Wang et al., 2017; Zhou et al., 2018; Gao et al., 2019; Wang et al., 2020b; Shao et al., 2020), Bcl-xL (Zhou et al., 2018; Wang et al., 2020b), Mcl-1 (Zhou et al., 2018; Wang et al., 2020b), and XIAP (Zhou et al., 2018; Wang et al., 2020b), thus inducing apoptosis-associated caspase activation.

##### Targeting the Extrinsic Pathway

The death receptor (DR)-mediated apoptosis signaling pathway is a kind of a signal pathway that interacts with proteins mediated by transmembrane receptors. Death receptors belong to the tumor necrosis factor receptor (TNFR) superfamily, including Fas (also known as CD95 or Apo-1), TNFR-1, TNFR-2, DR3, DR4, and DR5 (Tummers and Green, 2017). Up to now, the Fas/FasL signaling pathway is considered as the most crucial signaling pathway in death receptor-mediated apoptosis. The corresponding mechanism is as follows: FAS is activated after binding with FasL. The activated Fas receptor recruits Fas-associated death domain protein (FADD) through the interaction of death domain (DD). FADD can not only bind Fas through DD at the C-terminal but also recruit procaspase-8 through N-terminal death effector domain and finally form Fas receptor–FADD–procaspase-8 death-inducing signaling complex (DISC). With the increase of the local concentration of DISC, procaspase-8 can catalyze the formation of active caspase-8 in DISC and then activate the downstream caspase factor, thus initiating the apoptosis process dependent on caspase cascade reaction (Wajant, 2002; Singh et al., 2016). Integral in the regulation and initiation of death receptor-mediated activation of programmed cell death is caspase-8 (Tummers and Green, 2017). To escape exogenous apoptosis, cancer cells often downregulate the expression of death receptors by using antagonistic machinery (Friesen et al., 1997). Therefore, recovering Fas expression by drug intervention is anticipated to trigger cancer cell apoptosis.

It was observed that TSN could induce apoptosis by increasing the expression of Fas to inhibit hepatocellular carcinoma cells (He et al., 2010; Liu et al., 2016). In addition, for the ovarian cancer CAOV-3 and ES-2 cells, when treated with TSN, the expression of Fas and FasL increased firstly, subsequently followed by the activation of caspase-8 and caspase-3. After the administration of z-IETD-FMK and z-DEVE-FMK (the inhibitors of caspase-8 and caspase-3, respectively), the inhibitory effect of TSN on the proliferation of these cancer cells was reduced (Li et al., 2019). The increased expression of Fas, caspase-8, and caspase-3 was also exhibited in leukemia K562 cells after incubation with TSN. Tumor necrosis factor (TNF)-related apoptosis-inducing ligand (TRAIL) is a promising anti-cancer agent that belongs to the TNF superfamily (Ashkenazi et al., 1999). Many cancer cells remain resistant to receptor-mediated apoptosis—for instance, the TRAIL-mediated apoptotic resistance appears in non-small-cell lung carcinoma, and the TSN showed a sensitivity-enhanced ability for cancer cell apoptosis. The underlying apoptotic mechanisms involved the upregulation of death receptor 5 (DR5) mediated by CCAAT/enhancer binding protein homologous protein (Li et al., 2017). Consistently, the upregulated expression of DR5 was found in TRAIL inhibiting hepatocellular carcinoma.

##### Other Mechanisms of Apoptosis

Recently, intracellular calcium ion (Ca^2+^) has been regarded to play a vital role in cell apoptosis, and Ca^2+^ overload was considered as the common final pathway of all types of cell death (Rizzuto et al., 2003). Ca^2+^ can induce apoptosis through regulating apoptosis-related molecules, producing reactive oxygen species (ROS), and changing cell membrane permeability (Orrenius et al., 2003). Shi *et al.* reported that TSN had an inhibitory effect on neuroblastoma glioma NG108-15 cells. Since TSN cannot penetrate the lipid bilayer, it was found that TSN exerted the anti-cancer role by facilitating the Ca^2+^ channel of the cell membrane (Hu et al., 1997). In order to further confirm which Ca^2+^ channel was regulated by TSN, a series of physiological experiments was carried out. These findings showed that TSN could facilitate the L-type Ca^2+^ channel in the cell membrane, thus promoting extracellular Ca^2+^ influx. In addition, intracellular Ca^2+^ overload can directly induce apoptosis (Li and Shi, 2004). It was reported that TSN, at different concentrations, could cause different changes to the PC12 cells, and these distinctions were closely related with the increase of [Ca^2+^]_i_, which indicated that Ca^2+^ played an essential role in cell apoptosis (Li and Shi, 2005).

Moreover, TSN can trigger some different pathways to induce cancer cell apoptosis. In one study, TSN was proven as a candidate of novel anti-cancer drugs for glioblastoma, and the underlying mechanism was inducing estrogen receptor β- and p53-mediated apoptosis (Cao et al., 2016). In addition, the c-Jun N-terminal kinase (JNK) signaling pathway was also involved in TSN-induced apoptosis. In a representative study, TSN was found to induce apoptosis by suppressing the CDC42/MEKK1/JNK pathway in human promyelocytic leukemia HL-60 cells (Ju et al., 2013). Furthermore, the PI3K–Akt signaling pathway acted a crucial role in apoptosis, and excessive activation of PI3K/Akt signaling in cancer cells was involved in the development of multidrug resistance (Burris, 2013). It was observed that TSN could reverse the resistance of human breast cancer cells 4T1 to adriamycin by inhibiting the PI3K/Akt signaling pathway (Wang et al., 2018). Recently, the role of microRNAs (miRNAs) in the treatment of cancer by TSN was explored. The outcomes showed that TSN could inhibit glioma progression property and induce apoptosis *via* upregulating the expression of miR-608 and downregulating the expression of miR-608 targets, Notch1 and Notch2, in glioma (Wang Q. et al., 2020).

#### Effect of Toosendanin on Tumor Invasion and Metastasis

About 90% of cancer-related deaths are associated with metastatic diseases rather than primary tumors (Jafri et al., 2017). Emerging evidence showed that TSN could inhibit cancer invasion and metastasis (Pei et al., 2017; Wang et al., 2017; Luo et al., 2018; Wang Q. et al., 2020). The migration and invasion of tumor cells is a complex process of multiple factors, stages, and steps. It involves the process of epithelial–mesenchymal transition (EMT), the degradation of basement membrane and extracellular matrix, the decrease of tumor cell adhesion, and angiogenesis. Among these pathological processes, EMT acts a pivotal role, and its function is transforming the polarized epithelial cell into the mesenchymal cell phenotype. E-cadherin is a marker of epithelial cells, which maintains the integrity of tissue structure by regulating the adhesion reaction between cells and matrix or between cells. Mesenchymal proteins such as N-cadherin and vimentin are highly expressed with the decrease of E-cadherin expression, which collaboratively induces the EMT process (Sachdeva and Mo, 2010). In the transforming growth factor-β1 (TGF-β1)-induced EMT cell model, the inhibitory effect of TSN on A549 and H1975 lung cancer cell migration, invasion, and adhesion was evaluated by wound healing, transwell, and adhesion assays. The outcomes indicated that TSN could weaken the abilities mentioned above, decrease the expression of N-cadherin, vimentin, and p-ERK1/2, and increase the expression of E-cadherin. Besides these, TGF-β1-induced EMT biomarkers can be reversed by silence Snail. These results demonstrated that TSN significantly inhibited TGF-β1-induced EMT and migration, invasion, and adhesion through the ERK/Snail pathway in lung cancer cells (Luo et al., 2018). Consistently, in CAVO-3 human ovarian cancer cells, invasion and migration could be inhibited by TSN, followed by increased NF-κB and E-cadherin and decreased N-cadherin, vimentin, and Snail. The underlying mechanism may be related with the NF-κB/Snail pathway (Li et al., 2018b). Recently, the β-catenin pathway has been considered as the crucial signaling pathway involved in EMT and plays an important role in metastasis. TSN was confirmed to inhibit migration, invasion, and TGF-β1-induced EMT *via* miR-200a-mediated downregulation of the β-catenin pathway in SGC-7901 human gastric carcinoma cells (Wang et al., 2017). Furthermore, TGF-β1-induced EMT and morphological change in pancreatic cancer cells can be reversed through upregulating E-cadherin expression and decreasing vimentin, ZEB1, and Snail levels. The corresponding mechanism was involved with blocking the Akt/mTOR signaling pathway (Pei et al., 2017).

#### Synergistic Effect of Toosendanin in Chemotherapy

Since the majority of chemotherapy drugs have serious side effects, it is still urgent to take adjuvant therapy to reduce the dosage of chemotherapy drugs and improve their anti-tumor activity. As a representative chemotherapy drug, cisplatin is used to treat non-small cell lung cancer (NSCLC), but due to the development of resistance, the benefit has been limited. It was found that TSN can enhance cisplatin sensitization against non-small cell lung cancer cells through targeting Anxa4 in NSCLC cells, decreasing the combination of Anxa4 with ATP7A, and decreasing the extracellular efflux of platinum. The results showed that TSN could be a potential candidate in treating drug-resistant NSCLC cells (Zheng et al., 2018). Adriamycin (ADM) is an anti-cancer drug with a high frequency of use in the treatment of breast cancer. Nevertheless, some kinds of breast cancer or breast cancers under repeated ADM exposure develop a strong resistance to ADM, which limits its clinical efficacy. Wang *et al.* found that TSN can attenuate the resistance of human breast cancer cells to ADM by inhibiting the expression of ABCB1 and PI3K/Akt signaling (Wang et al., 2018). Doxorubicin, another anti-cancer antibiotic, possesses a strong anti-tumor effect. However, the toxicity of high-dose doxorubicin is severe, especially to the heart. In MDA-MB-435 breast cancer cells, cotreatment of doxorubicin with TSN can significantly enhance the anti-breast cancer activity, and the mechanism may depend on the FoxO1-Bim/Noxa pathway.

#### Selective Inhibition Effect of Toosendanin on Tumor Cells

The specific inhibitory effect on tumors is crucial for the development of new anti-tumor drugs. In one study, Li *et al.* showed that TSN selectively increased the sensitivity of NSCLC cells, but not normal cells (293T, BEAS-2B, HBE, L02, and PBMC), to TRAIL-induced apoptosis (Li et al., 2017). For the toxicity of hepatocellular carcinoma, after treating with toosendanin (0.5 µM) for 72 h, the inhibition rate for SMMC-7721 and Hep3B cells was 56 and 42%, respectively, and for IAR-20 and L02 cells (normal liver cell lines), it was 21 and 20%, respectively (He et al., 2010). When treated with a lower dosage range (0.25–1 µM), toosendanin could selectively kill human gastric carcinoma cells (MGC-803, BGC-823, HGC-27, AGS, SGC-7901, and MKN-45) rather than normal human gastric epithelial cells (GES-1) (Wang et al., 2017). The selective inhibition effect of toosendanin against tumor cells was cross-validated in different research groups. According to the results of Yan and Zhang, the IC_50_ of toosendanin on normal primary hepatocyte cells was 30.65 and 14.94 μM, respectively, which was higher than its suppressing effect on hepatocellular carcinoma (0.5–0.9 μM) as reported by other teams (Zhang et al., 2008; He et al., 2010; Liu et al., 2016; Yan et al., 2019).

#### Anti-tumor Effects of Other Limonoids

Inspired by the remarkable bioactivity, numerous works have been conducted to isolate and identify the potential lead compounds from genus *Melia* plants for cancer treatment. Three limonoids, including trichilinin B, 3-deacetyl-4′-demethyl-28-oxosalannin, and 23-hydroxyohchininolide, were cytotoxic to gastric cancer cell line of AZ521 with IC_50_ values of 58.2, 3.2, and 78.5 µM, respectively (Pan et al., 2014b). In another study, 31 limonoids were isolated and identified from the fruits of *M. azedarach*; among them, several compounds including meliarachin C, 12-dehydroneoazedarachin D, salannin, 1-*O*-decinnamoyl-1-*O*-benzoylohchinin, ohchininolide, 1-*O*-decinnamoyl-1-*O*-benzoylohchininolide, 23-hydroxyohchininolide, 1-*O*-decinnamoyl-1-*O*-benzoyl-23-hydroxyohchininolide,1-*O*-decinnamoyl-1-*O*-benzoyl-28-oxoohchinin, mesendanin E, 1-benzoyl-1-detigloylohchinolal, and nimbolinin D possessed anti-tumor effect against AZ521 cells, and 12-dehydroneoazedarachin D showed the most potent cytotoxicity with an IC_50_ value of 11.8 µM (Akihisa et al., 2013). In addition, trichilinin E exhibited apparent cytotoxicity against the gastric cancer cell line SGC-7901 (Zhou et al., 2016).

For lung cancer treatment, 12-hydroxyamoorastatone, 12-hydroxyamoorastatin, 12-acetoxyamoorastatin, meliarachin C, 1-*O*-decinnamoyl-1-*O*-benzoylohchinin, 1-*O*-decinnamoyl-1-*O*-benzoyl-23-hydroxyohchininolide, and 1-benzoyl-1-detigloylohchinolal were reported to be toxic against A549 cell, and 1,12-diacetyltrichilin B showed significant cytotoxicity with an IC_50_ value of 0.93 µM (Tada et al., 1999; Zhao et al., 2012; Akihisa et al., 2013). The isolated limonoids can also inhibit colorectal cancers. Among them, 12-hydroxyamoorastatone, 12-hydroxyamoorastatin, and 12-acetoxyamoorastatin displayed obvious antitumor activity against HCT-15 cell. Notably, 12-acetoxyamoorastatin possessed stronger cytotoxicity than antimycin A (positive drug), which indicated that it could be a candidate in curing colorectal cancer (Ahn et al., 1994). The anti-tumor effect was also found in SW480 colorectal cancer cells by 1,12-diacetyltrichilin B (Yuan et al., 2013). It was reported that lots of limonoids have a cytotoxic effect on leukemia HL-60 cells. Among them, 3-deacetyl-4′-demethyl-28-oxosalannin, 1,12-diacetyltrichilin B, meliarachin C, 1-*O*-cinnamoyltrichilinin, 23-methoxyohchininolide A, 1-benzoyl-1-detigloylohchinolal, 1-cinnamoyl-1-detigloylohchinolal, and nimbolinin D were considered as the most potential compounds with an IC_50_ value lower than 10 µM (Zhao et al., 2012; Akihisa et al., 2013; Pan et al., 2014b). Simultaneously, 12 limonoids from the fruits of *M. azedarach* also possessed marked anti-tumor activity against breast cancer MCF-7 cell with IC_50_ values from 0.06 to 94.8 µM (Akihisa et al., 2013). Toosendansin H, meliatoxin B1, and 1,12-diacetyltrichilin B, which were isolated from the fruits and stem bark of *M. toosendan*, were toxic to MCF-7 cell (Zhao et al., 2012; Li et al., 2020b). Moreover, trichilinin B and 21-hydroxyisoohchininolide can inhibit the growth of SK-BR-3 cell, which is another type of breast cancer cell (Pan et al., 2014b).

MTT assay was applied to evaluate the cytotoxicity of nine limonoids against lymphoma P388 cell. The outcomes revealed that all the nine limonoids possessed apparent toxicity, of which 12-deacetyltrichilin I, 3-deacetyltrichilin H, and trichilin D exhibited significant bioactivity with IC_50_ values of 0.011, 0.045, and 0.055 μg/ml, respectively (Takeya et al., 1996b). In the treatment of oral epithelial carcinoma, trichilin H (IC_50_ = 0.11 μg/ml) and 12-*O*-methylvolkensin (IC_50_ = 8.72 μg/ml) were observed to be toxic to kB cells (Tada et al., 1999). Zhou *et al.* isolated three new C-seco limonoids and one new tetracyclic limonoid from the fruits of *M. azedarach*, and their cytotoxicity against cervical cancer HeLa S3 cells was assessed. The results showed that 15-*O*-deacetylnimbolidin B and 12-*O*-deacetyltrichilin H had a strong inhibitory effect, while 15-*O*-deacetyl-15-*O*-methylnimbolidin A and 15-*O*-deacetyl-15-*O*-methylnimbolidin B had weak cytotoxicity (Zhou et al., 2005). Furthermore, using bioassay-guided chemical investigation, 12-hydroxyamoorastatone, 12-hydroxyamoorastatin, and 12-acetoxyamoorastatin were isolated, and the anti-tumor effects were evaluated. Three compounds showed potent cytotoxicity against human malignant melanoma SK-MEL-2 and human CNS carcinoma XF498 (Ahn et al., 1994). Several limonoids also possessed noticeable hepatotoxicity. Among them, 12-ethoxynimbolinins G and 1,12-diacetyltrichilin B were toxic to SMMC-7721 cells, with IC_50_ values of 27.6 and 0.36 µM, respectively, and trichilinin E had cytotoxicity against HepG2 cell (Yuan et al., 2013; Zhou et al., 2016; Zhang et al., 2018).

#### Structure–Activity Relationships With Anti-tumor

In order to further explain the effect of different skeletons and substituents on the anti-tumor activities of limonoids, the corresponding structure–activity relationships (SAR) were systematically summarized. For trichilin-type limonoids, it was found that meliarachin C and toosendanin with a β-oriented epoxy ring at C-14/C-15 possessed a significant bioactivity than those of their 15-oxo analogues (meliarachin K and meliarachin G) against HL60 and AZ521 cells. Meanwhile, the inhibitory effect of 15-oxo trichilins against HL60 and AZ521 cells could be enhanced by deacetoxylation at C-12 (12-dehydroneoazedarachin D *vs*. meliarachin K). For vilasanin-type limonoids, acetylation of the hydroxy group at C-3 was able to strengthen the anti-cancer effect against HL60 cells—for instance, 1-*O*-cinnamoyltrichilinin (IC_50_ = 5.8 μM) *vs*. trichilinin D (IC_50_ = 61.2 μM). This structural modification also enhanced the activity against HL60 cells in the salannin-type limonoids with a cinnamoyl group at C-1 (ohchinin acetate *vs*. ohchinin). By contrast, acetylation at C-3 could reduce the bioactivity of salannin-type limonoids, which bore a tigloyl group at C-1 (salannin *vs*. 3-*O*-deacetylsalannin). In addition, the substitution of a methacrylic group at C-1 with a benzoyl group reduced the activity against HL60 and AZ521 cells (3-deacetyl-4′-demethyl-28-oxosalannin *vs*. 1-*O*-decinnamoyl-1-*O*-benzoyl-28-oxoohchinin). The characteristic SAR was also found in ohchinolal-type limonoids. The study showed that the inhibitory properties against HL60 cells would be reduced when the cinnamoyl group at C-1 was substituted by a methacrylic or a tigloyl group (1-cinnamoyl-1-detigloylohchinolal *vs*. mesendanin E and ohchinolal) (Akihisa et al., 2013). Surprisingly, the demethacrylation of mesendanin E and the detigloylation of ohchinolal at C-1 were able to enhance the anti-cancer ability against HL60, A549, and AZ521 cells. This finding indicated that substitution of methacrylic or tigloyl group at C-1 was probably related with anti-cancer property (Pan et al., 2014a) (Figures 10).

**FIGURE 10:**
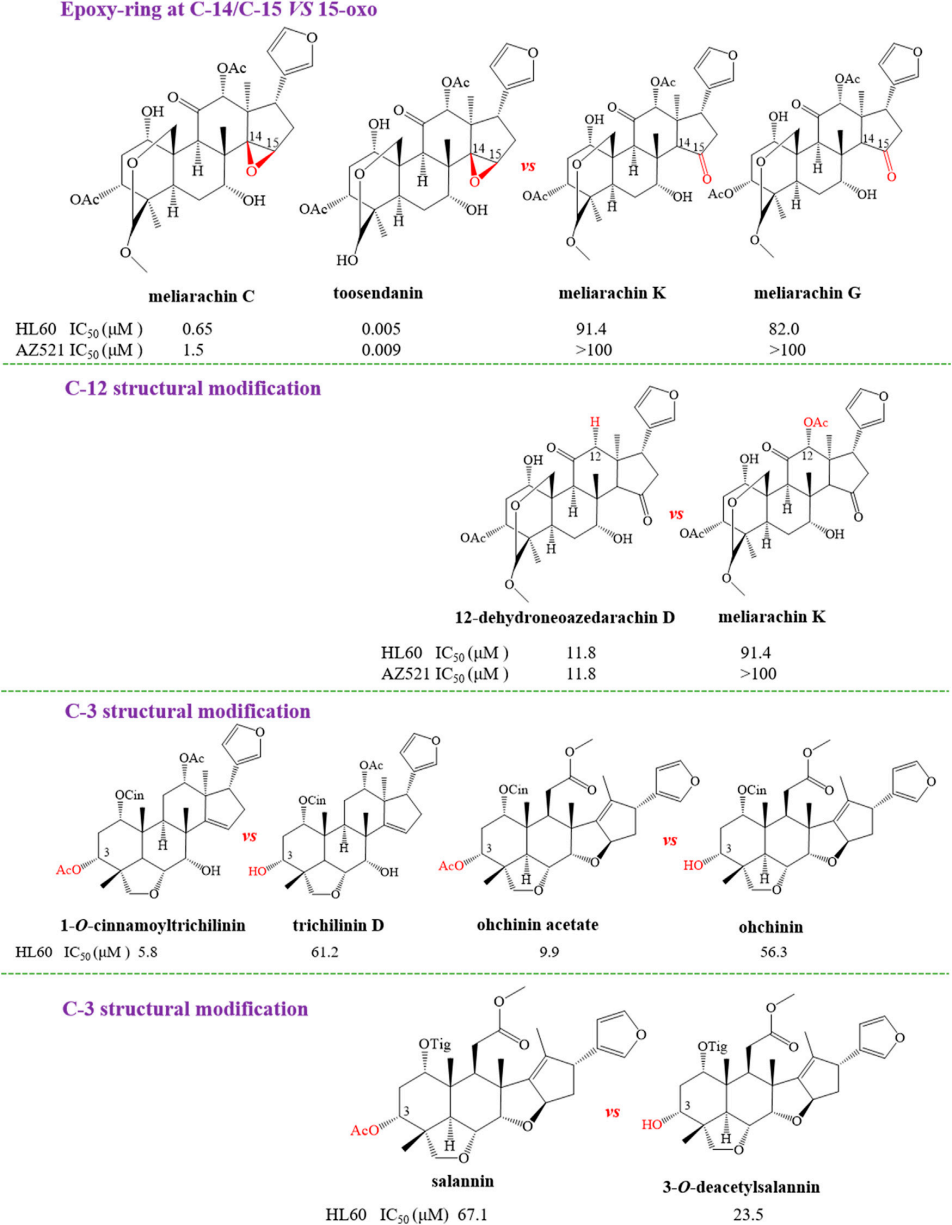
The anti-tumor structure–activity relationship of limonoids.

**FIGURE 10:**
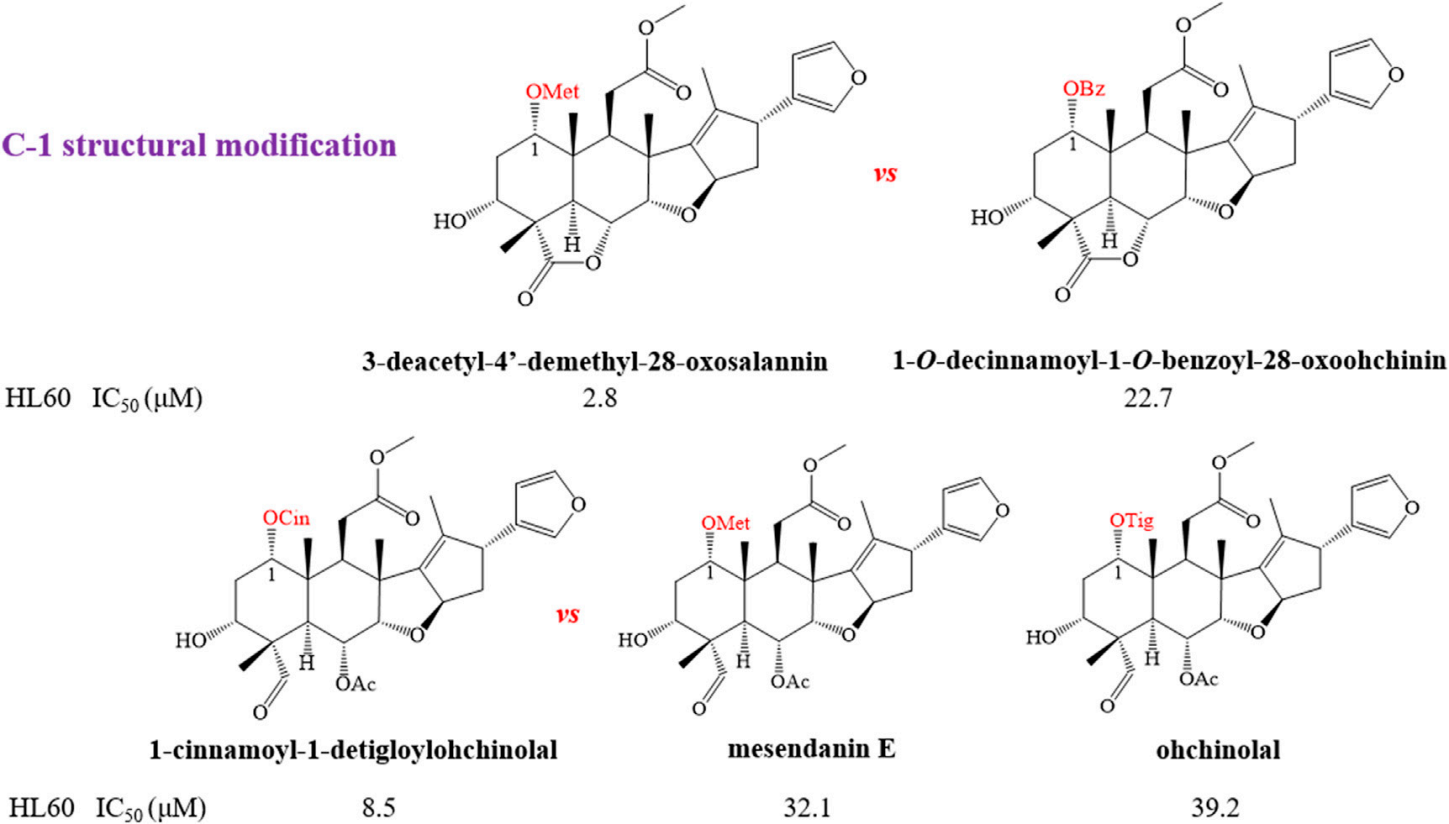
(continue) The anti-tumor structure—activity relationship of limonoids.

### Antifeeding and Insecticide Effects

#### Antifeeding and Insecticide Effects of Toosendanin

*M. toosendan* and *M. azedarach*, two common agricultural insecticidal and *Ascaris*-repellent Chinese herbs, have been recorded in ancient China, and TSN has been considered as the main active compound for insecticidal effect. The content of TSN in *M. toosendan* is high, and it is environmentally friendly due to its botanical property (Shi and Li, 2007). Recently, TSN showed distinguished potential in the field of agricultural anthelmintic. It was reported that TSN possessed remarkable repellent and insecticidal effects on *Pieris rapae*, and the underlying mechanism has been explored comprehensively. TSN also has obvious antifeedant activity. TSN at different dosages can affect the feeding reaction and feeding speed of *P. rapae*, which exhibited different poisoning symptoms. A high dose of TSN can induce the paralysis and coma of *P. rapae* and cause pathological changes of the midgut. Meanwhile, a low dose of TSN can transform the larvae into abnormal insects. One study was designed to evaluate the effects of TSN on the activities of several important enzymes in *P. rapae*. The results showed that TSN was able to reduce the activities of microsomal multifunctional oxidase, protease, and intestinal acetylase, which indicated that the interference of TSN on the detoxification enzymes of *P. rapae* may be responsible for the toxic reaction (Zhang and Zhao, 1992a). In addition, TSN was capable of causing severe damage to midgut tissue, resulting in abscission and autolysis (Zhang and Zhao, 1991). Furthermore, the physiological metabolism of *P. rapae* was disturbed by TSN, and abnormal biological oxidation was induced *in vivo*, which demonstrated that TSN could inhibit the central nervous system of the larva (Zhang et al., 1992b).

Apart from *P. rapae*, TSN possessed potent growth inhibition on other species of insects—for instance, TSN possessed strong stomach toxicity against *Mythimna separate* (LC_50_ = 252.23 μg/ml), and this process was located and targeted on the microvilli of the midgut cells. Subsequently, TSN induced the destruction of midgut epithelial cells, which caused the regurgitation, paralysis, and death of *M. separate* (Li H. et al., 2020). Another research reported that TSN could disrupt yolk deposition in oocytes, blood ingestion and digestion, and ovary ecdysteroid production in *Aedes aegypti* (Ma et al., 2013). Huang *et al.* found that the activities of pepsase and tryptase in *Brachionus plicatilis* can be suppressed by TSN, which may be responsible for the feeding deterrent property (Huang et al., 2017). In order to solve the problems of biological pollution in the cultivation scale of microalgae, TSN was selected to conduct an insecticidal test. The outcomes revealed that TSN possessed obvious toxicity to *Stylonychia mytilus* (a type of ciliate), while it is relatively safe to *Chlorella pyrenoidosa* (a type of microalgae) (Xu et al., 2019). Moreover, TSN was reported to inhibit the growth of *Sirophilus oryzae* and *Cryptolestes ferrugineus*, two common store-product insects (Xie et al., 1995). In addition to the control of agricultural pests, TSN has become a clinical anti-*Ascaris* drug in China since the 1950s (Shi and Li, 2007).

#### Antifeeding and Insecticide Effects of Other Limonoids

The insect antifeedant activities of other limonoids from genus *Melia* plants have been extensively evaluated, and the structure–activity relationships were also discussed. By using the conventional leaf disk method, the antifeedant activity against *Spodoptera eridania* was tested for ohchinolide C, salannin, nimbolidin B, nimbolidin C, nimbolidin D, nimbolidin E, and nimbolidin F. The outcomes showed that these compounds, at a concentration of 500 ppm, can significantly affect feeding (Nakatani et al., 1996; Zhou et al., 1997), while trichilinin B, trichilinin C, ohchinolide B, ohchinolide C, and 3-*O*-acetylohchinolal exhibited a weak antifeedant activity against *S. eridania* at a concentration of 1,000 ppm (Nakatani, 1999). At the concentration of 400 ppm, 12-*O*-acetylazedarachin A, 12-*O*-acetylazedarachin B, trichilin H, trichilin I, trichilin J, trichilin K, and trichilin L have an inhibitory effect on the feeding amounts of leaf compared to the controls (Nakatani, 1999). It was found that 12-hydroxyamoorastatone, azedarachin A, trichilin B, and 12-hydroxyamoorastatin displayed a potential antifeedant activity with MIC values of 250, 200, 200, and 150 ppm, respectively (Zhou et al., 1996; Nakatani, 1999). At the same time, the most potent antifeedant limonoids were found to be meliacarpinin A, meliacarpinin C, and meliacarpinin D, with a MIC value of 50 ppm (Nakatani, 1999). In addition, some studies focused on discovering the effective antifeedants against *Spodoptera littoralis*, another kind of plant pest. As a result, nimbolinin A, nimbolinin B, nimbolinin C, nimbolinin D, trichilinin D, trichilinin E, 1-*O*-cinnamoyltrichilinin, 1-deacetylnimbolinin A, spirosendan, and 2-deacetylnimbolinin B exhibited a weak activity at 1,000 ppm (Nakatani et al., 2000). The MIC value of neoazedarachin A, neoazedarachin B, neoazedarachin D, 13-*O*-acetylazedarachin A, and 1-acetyltrichilin H against *S. littoralis* was 400 ppm, and the MIC value for isotoosendanin, TSN, and azedarachin B was 300, 200, and 200 ppm, respectively (Nakatani, 1999; Nakatani et al., 2000). Furthermore, 1-cinnamoyl-3-acetyl-11-hydroxymeliacarpin was found to possess an attractive insecticidal activity with a LC_50_ value of 0.48 ppm and a EC_50_ value of 0.27 ppm (Bohnenstengel et al., 1999). LC–MS guided isolation method was applied to screen promising antifeedant compounds against the fifth-instar larvae of *P. rapae*, and TSN, isotoosendanin, 12α-hydroxyamoorastatone, mesendanin H, meliatoosenin E, 3-*O*-acetylohchinolal, salannin, and ohchinal were found to inhibit the feeding ability of *P. rapae*. Among these compounds, mesendanin H was proven to be the most active limonoid, with an AFC_50_ value of 0.11 mM (Wang et al., 2020a). The antifeedant activity against *Spodoptera exigua* of azedarachin A, 12-*O*-acetylazedarachin A, 12-*O*-acetylazedarachin B, and 1-deoxy-3-tigloyl-11-methoxymeliacarpinin was proven, and the MIC values ranged from 200 to 400 ppm (Huang et al., 1994). For the purpose of finding novel naturally occurring insecticides from plants, Carpinella *et al.* evaluated the antifeedant and insecticide properties of 12α-hydroxyamoorastatone and azadirachtin against *Epilachna paenulata* by choice and no-choice assays. In a choice-assay study, 12α-hydroxyamoorastatone and azadirachtin inhibited feeding with ED_50_ values of 0.80 and 0.72 ug/cm^2^. These two compounds both possessed a remarkable insecticide activity, which was evidenced by low food intake, weight loss, and even significant mortality (LD_50_ values of 0.76 and 1.24 μg/cm^2^) (Carpinella et al., 2003).

#### Structure–Activity Relationships With Insecticidal Effect

**FIGURE 11:**
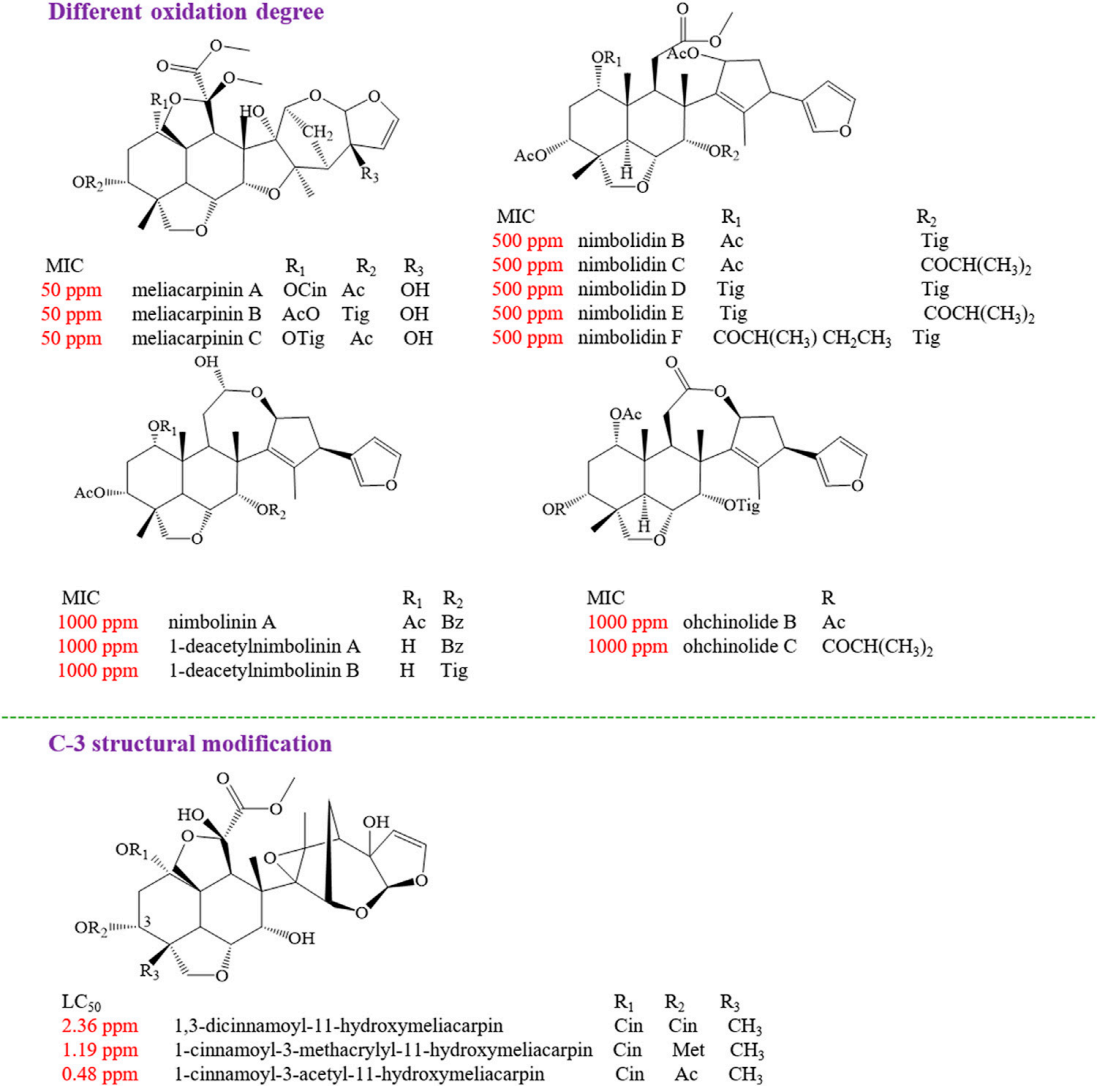
The insecticidal structure–activity relationship of limonoids

The structure–activity relationships of limonoids have attracted extensive interest for their remarkable insecticidal activity. It was found that the highly oxidized C-seco limonoids, such as meliacarpinin-type limonoids (meliacarpinin A, meliacarpinin C, and meliacarpinin D), exhibited the most potent antifeedant activity than the less oxidized types of C-seco limonoids, such as nimbolinins (nimbolinin A and 1-deacetylnimbolinin A-B), ohchinolides (ohchinolide B-C), nimbolidins (nimbolidin B-F), and salannin. Apart from meliacarpinins, the intact trichilin-type limonoids with C-19/C-29 bridged acetals were considered as the higher active compounds, of which 12-hydroxyamoorastatin showed the most potent activity (Nakatani, 1999). In another study, the most active compound against *S. eridania* was azedarachin A with a 12-OH function (Zhou et al., 1996). The insecticidal activity evaluation of three meliacarpins (1,3-dicinnamoyl-11-hydroxymeliacarpin, 1-cinnamoyl-3-methacrylyl-11-hydroxymeliacarpin, and 1-cinnamoyl-3-acetyl-11-hydroxymeliacarpin) suggested that the property of the ester substituent at C-3 acted a critical role for the insecticidal effect. The results showed that 1-cinnamoyl-3-acetyl-11-hydroxymeliacarpin that bore a small and relatively hydrophilic acetyl group at C-3 possessed a high activity, while 1,3-dicinnamoyl-11-hydroxymeliacarpin with a bulky and more lipophilic cinnamoyl group exhibited the lowest activity (Bohnenstengel et al., 1999) (Figure 11).

### Anti-botulinum Effect

Botulinum neurotoxin (BoNT), the strongest known biotoxin in the world, is a kind of toxin secreted by *Clostridium botulinum*, which includes seven distinct serotypes (identified as A to G). The nerve endings of a cholinergic neuromuscular junction are considered as its key targets. Botulinum neurotoxin can inhibit the release of acetylcholine through the enzymatic hydrolysis of SNARE protein, which plays a crucial role in neurotransmitter release. Then, the signal transmission of neuromuscular connection was further blocked to paralyze the muscle, which eventually led to the death of animals due to respiratory failure (Rossetto et al., 2014). The four-step action hypothesis, the proposed mechanism of botulinum toxin on target cells, demonstrated that botulinum toxin binds to receptors on the cell surface firstly and enters the nerve cell through receptor-mediated internalization. Subsequently, acidification of endocytic vesicles caused the translocation of light chains from the vesicles to the cytoplasm, and the light chains cleave the SNARE protein in the cytosol, thereby completing the last step of the intracellular action of the toxin (Humeau et al., 2000). TSN has been proven to block neuromuscular connection in the phrenic nerve–diaphragm specimens of rats, which was similar to that of BoNT in many aspects—for instance, the dosage–response relationship, the higher temperature coefficient, and the irreversibility of the blocking effect existed in both substances when blocking neuromuscular connections. Nevertheless, there are some differences between TSN and BoNT—for instance, TSN can cause submicroscopic changes of presynaptic motor nerve endings, which refer to the reduced number of synaptic vesicles and the widened synaptic cleft (Xiong, 1985). It was noteworthy that TSN has a facilitation phase before the final inhibition of neurotransmitter release. It means that the quantum release of acetylcholine by TSN is a biphasic regulation of facilitation followed by inhibition (Shi et al., 1982).

Interestingly, TSN, which has similar bioactive properties with BoNT, unexpectedly possesses a prominent anti-botulism effect. Early in the 1980s, TSN was proven to have an obvious therapeutic effect on BoNT-poisoned animals. In the isolated phrenic nerve and diaphragm muscle specimens of mice, both the TSN and BoNT administered simultaneously or TSN administered beforehand showed a significant antagonistic effect on BoNT (Li and Sun, 1983). The anti-botulism effect of TSN exhibited the properties of fast onset and long action time in the isolated neuromuscular specimens and rat models. In addition, TSN has a therapeutic effect on various types of botulism, such as A, B, and E, in mice and rhesus monkeys, which showed broad-spectrum anti-botulism effects (Janda, 2008). Furthermore, in a clinical study, oral administration of TSN can relieve symptoms of botulism, and the patients will recover when treated with the combination of TSN and anti-botulinum serum (Zou et al., 1985). Undoubtedly, structure–activity relationships attracted enough attention in drug research and development. Whether the unusual AB-ring of TSN is related with its anti-botulinum properties was tested. A synthetic strategy allowing access to the AB-fragment of TSN was achieved from a *trans*-decalin system. However, none of these synthetic structures exhibited antagonism against BoNT/A in the rat spinal cord cellular assay, which demonstrated that complete ABCD nucleus was essential for the anti-BoNT effect of TSN (Nakai et al., 2010).

Recently, the mechanism study revealed that TSN was capable of endowing neuromuscular junctions with a high tolerance to BoNT. Preincubation with TSN for 5 min can enhance the tolerance to BoNT/A in the isolated phrenic nerve and diaphragm muscle specimens of mice (Li and Sun, 1983). In another study, rat phrenic nerve–diaphragm specimens that were preincubated with TSN showed apparent antagonism to botulism, which was manifested as significantly delayed at the time of neuromuscular transmission inhibition. It was found that the anti-BoNT effect of TSN was closely related with the facilitation of neurotransmitter release induced by TSN in a time-dependent manner. In one research, rats injected with TSN (7 mg/kg, i.h.) or blank solvent were sacrificed at different time points (from 15 min to several days). The results showed that the rat phrenic nerve–diaphragm specimens exhibited high BoNT tolerance after injection with TSN, and the frequency of miniature end-plate potentials was higher than that of the control (Shih and Hsu, 1983).

In the light of the four-step action hypothesis, cleaving of SNARE protein by the light chains was considered as the final process to activate a toxic effect, therefore inhibiting the cleaving procedure emerges as a potential therapeutical strategy. Inspired by this tactic, Zhou *et al.* explored the anti-BoNT mechanism of TSN by Western blotting. Surprisingly, TSN treatment did not affect the expression of synaptosomal-associated protein of 25 kDa (SNAP-25), syntaxin, and synaptobrevin/vesicle-associated membrane protein in rat cerebral synaptosomes. However, it can completely antagonize the cleavage of SNAP-25 induced by BoNT/A. When BoNT acts directly on synaptosomes, TSN can still partially antagonize the cleavage of SNAP-25, but it cannot inhibit the cleavage of SNAP-25 on the synaptosome membrane by light-chain BoNT/A. Thus, the underlying mechanism of TSN against BoNT/A is to block the approach of BoNT to its enzyme substrate (Zhou et al., 2003).

As we know, cleaving of SNARE protein by light chains is a critical event in botulism, and the heavy-chain-formed channel has been confirmed to be indispensable in the translocation of the light chain into the cytosol (Blaustein et al., 1987). To verify the effect of TSN on the channel-forming activity of BoNT/A, the inside-out single-channel recording patch-clamp technique was applied to record the BoNT/A-induced currents in the presence and absence of TSN. The results showed that TSN administration could delay the channel formation and reduce the size of the channels in the PC12 cell membrane, which indicated that TSN could inhibit BoNT/A by interfering with the translocation of BoNT *via* inhibiting its pore-forming activity (Li and Shi, 2006).

### Anti-inflammatory Effect

Limonoids from genus *Melia* were considered as an emerging source for discovering potential anti-inflammatory lead compounds. In a representative research, the anti-inflammatory effect of TSN was found in dextran sulfate sodium-induced colitis model. After treatment with TSN, the secretion of proinflammatory cytokines and oxidative stress were blocked, and M1 macrophage polarization and the activation of NLR family pyrin domain containing 3 inflammasome were restrained, accompanied with upregulation of HO-1/Nrf2 expression (Fan et al., 2019). In addition, acetic acid-induced vascular permeability, λ-carrageenan-induced hind paw edema tests, and acetic acid-induced writhing and hot-plate tests were applied to evaluate the anti-inflammatory effect of limonoids. As a result, two limonoids, iso-toosendanin and 1-*O*-tigloyl-1-*O*-debenzoylohchinal, isolated from the fruit of *M. toosendan* were proven to have anti-inflammatory and analgesic effects (Xie et al., 2008). The NF-κB pathway plays a critical role in inflammation, and one report demonstrated that 1-*O*-tigloyl-1-*O*-deacetyl-nimbolinin B (TNB) possessed the most obvious anti-inflammatory effect than the other two limonoids, including nimbolinin A and nimbolinin B, and the underlying mechanism was through suppressing NF-κB and JNK activation in microglia cells. In this study, administration with TNB can reduce the production of nitric oxide (NO) and TNF-α in lipopolysaccharide (LPS)-stimulated microglia cells (Tao et al., 2014). In another research, NF-κB luciferase assay was applied to rapidly screen potential NF-κB regulators from the fruits of *M. toosendan*. As a result, seven limonoids were found to inhibit NF-κB activation at the concentration of 10 μM (Zhu et al., 2014).

In the LPS-induced mouse macrophage RAW 264.7 cell inflammatory model, five limonoids, including trichilinin B, 3-deacetyl-28-oxosalannolactone, ohchinin, 23-hydroxyohchininolide, and 21-hydroxyisoohchininolide, exhibited a significant anti-inflammatory activity by inhibiting the production of NO with IC_50_ values from 28.7 to 87.3 μM. Meanwhile, all these five compounds displayed no or almost no toxicity to the cells (Pan et al., 2014b). Toosendane B and toosendane C, another two limonoids from the bark of *M. toosendan*, can also obviously reduce NO production in RAW 264.7 cells induced by LPS (Hu et al., 2018). Four NO-inhibited limonoids, including ohchinin, salannin, nimbolinin D, and mesendanin E, were isolated from *M. azedarach*, of which salannin was found to inhibit the expression levels of iNOS and COX-2 proteins in a concentration-dependent manner (Akihisa et al., 2017). In addition, meliazedalides B, a trichilin-class limonoid from the fruits of *M. azedarach*, also showed a weak anti-inflammatory effect (Qiu et al., 2019). In order to screen anti-neuroinflammatory compounds from *M. azedarach*, a LPS-induced microglia BV-2 cell model was established. Seven compounds, including 1-*O*-benzoyl-3-*O*-deactylnim-bolinin C, 3-deacetyl-12-*O*-methylvolkensin, 12-*O*-methyl-1-*O*-deacetyl-nimbolinin, trichilinin B, 1-*O*-cinnamoyltrichilinin, 1-acetyltrichilinin, and 12α-hydroxyamoorastatone, were isolated and confirmed as potential inhibitors of NO production, with IC_50_ values from 7.73 to 26.75 μM (Park et al., 2020).

### Antibacterial and Antifungal Effects

Recently, several limonoids were found to possess a potential antibacterial activity. In the year 2007, 10 limonoids were isolated from the fruits of *M. toosendan*, among which three compounds, including 12-ethoxynimbolinins C, 1-*O*-cinnamoyltrichilinin, and trichilinin B, were validated to have an antibacterial activity against oral pathogen *Porphyromonas gingivalis*, with MIC values of 15.6, 31.3, and 31.5 μg/ml, respectively. Nevertheless, they exhibited no obvious bioactivity against *Streptococcus mutans*. The results indicated that these three limonoids might be specific inhibitors of *P. gingivalis* (Zhang et al., 2007). In 2016, this research group found that 1α,7α-ditigloyloxy-3α-acetoxyl-12α-ethoxylnimbolinin and 1α-tigloyloxy-3α-acetoxyl-7α-hydroxyl-12β-ethoxylnimbolinin, the other two new limonoids, possessed antibacterial activity against *P. gingivalis*, with MIC values of 15.2 and 31.25 μg/ml, respectively (Zhang et al., 2016). Unlike previously reported limonoids from fruits or bark, three limonoids were isolated from the seeds of *M. azedarach*, and 7-cinnamoyltoosendanin had significant inhibitory effects against *Micrococcus luteus* and *Bacillus subtilis*, with MIC values of 6.25 and 25 μg/ml, respectively (Liu et al., 2011). By using a microdilution assay, Su *et al.* determined the antibacterial activity of meliarachin D and meliarachin H. The results showed that meliarachin D can inhibit both *Staphylococcus aureus* (MIC = 50 μg/ml) and *B. subtilis* (MIC = 50 μg/ml); in contrast, meliarachin H can only suppress the growth of *B. subtilis*, with a MIC value of 25 μg/ml (Su et al., 2011).

### Other Pharmacological Effects

Apart from the pharmacological effects mentioned above, limonoids also possessed some other bioactivities. In 2018, TSN was reported to exhibit anti-obesity property. By using Oil Red O staining assay, TSN could attenuate lipid accumulation in preadipocytes 3T3-L1. In addition, treatment with TSN can also decreased the mRNA and protein levels of adipocytokines (adiponectin and leptin), CCAAT/enhancer-binding proteins α, peroxisome proliferator-activated receptor γ, fatty acid synthase, and acetyl-CoA carboxylase in adipocytes. Moreover, the weight of gonadal white fat and the serum triacylglycerol content in high-fat-diet-fed mice were reduced by administration with TSN. The underlying mechanism is through activating the Wnt/β-catenin pathway (Chen et al., 2018). One study showed that, through altering polymerase acidic protein nuclear localization, TSN could inhibit early influenza A virus infection (Jin YH. et al., 2019). Similarly, hepatitis C virus infection was also inhibited by TSN. It can enhance the effect of alpha interferon (α-IFN), a classical drug in the treatment of HCV infection, and upregulate the expression of α-IFN-related protein (Watanabe et al., 2011). The pharmacological effects are summarized in Table 2.

**TABLE 2:** Pharmacological effects of limonoids.

Compounds	Models		Treatment	*In vitro/vivo*	Effects	Reference
***Anti-tumor effect***	—					
**Gastric cancer**	—					
Toosendanin	MKN-45 cells		60–100 nM	*In vitro*	Inducing apoptosis	Shao *et al*. (2020)
Toosendanin	AGS cells		0.5–2 μM	*In vitro*	Inhibiting cell proliferation and inducing apoptosis	Zhou *et al*. (2018)
Toosendanin	HGC-27 cells		0.1–0.5 μM	*In vitro*	Inhibiting cell proliferation and inducing apoptosis	Zhou *et al*. (2018)
Toosendanin	SGC-7901 cells		0.25–4 µM	*In vitro*	Inhibiting proliferation, invasion, migration and inducing apoptosis	Wang *et al*. (2017)
Toosendanin	MGC-803 cells		30–70 nM	*In vitro*	Inhibiting cell proliferation	Liu *et al*. (2019)
Toosendanin	HGC-27 cells		0.6 µM	*In vitro*	Inhibiting cell proliferation and invasion	Liu *et al*. (2019)
Trichilinin B	AZ521 cells		IC_50_ = 58.2 µM	*In vitro*	Cytotoxicity against AZ521 cells	Pan *et al*. (2014b)
3-Deacetyl-4′-demethyl-28-oxosalannin	AZ521 cells		IC_50_ = 3.2 µM	*In vitro*	Inducing apoptosis	Pan *et al*. (2014b)
23-Hydroxyohchininolide	AZ521 cells		IC_50_ = 78.5 µM	*In vitro*	Cytotoxicity against AZ521 cells	Pan *et al*. (2014b)
Meliarachin C	AZ521 cells		IC_50_ = 1.5 µM	*In vitro*	Cytotoxicity against AZ521 cells	Akihisa *et al*. (2013)
Toosendanin	AZ521 cells		IC_50_ = 0.009 µM	*In vitro*	Cytotoxicity against AZ521 cells	Akihisa *et al*. (2013)
12-Dehydroneoazedarachin D	AZ521 cells		IC_50_ = 11.8 µM	*In vitro*	Cytotoxicity against AZ521 cells	Akihisa *et al*. (2013)
Salannin	AZ521 cells		IC_50_ = 55.4 µM	*In vitro*	Cytotoxicity against AZ521 cells	Akihisa *et al*. (2013)
1-*O*-Decinnamoyl-1-*O*-benzoylohchinin	AZ521 cells		IC_50_ = 82.3 µM	*In vitro*	Cytotoxicity against AZ521 cells	Akihisa *et al*. (2013)
Ohchininolide	AZ521 cells		IC_50_ = 82.9 µM	*In vitro*	Cytotoxicity against AZ521 cells	Akihisa *et al*. (2013)
1-*O*-decinnamoyl-1-*O*-benzoylohchininolide	AZ521 cells		IC_50_ = 34.7 µM	*In vitro*	Cytotoxicity against AZ521 cells	Akihisa *et al*. (2013)
23-Hydroxyohchininolide	AZ521 cells		IC_50_ = 78.5 µM	*In vitro*	Cytotoxicity against AZ521 cells	Akihisa *et al*. (2013)
1-*O*-Decinnamoyl-1-*O*-benzoyl-23-hydroxyohchininolide	AZ521 cells		IC_50_ = 55.7 µM	*In vitro*	Cytotoxicity against AZ521 cells	Akihisa *et al*. (2013)
1-*O*-Decinnamoyl-1-*O*-benzoyl-28-oxoohchinin	AZ521 cells		IC_50_ = 61.7 µM	*In vitro*	Cytotoxicity against AZ521 cells	Akihisa *et al*. (2013)
Mesendanin E	AZ521 cells		IC_50_ = 80 µM	*In vitro*	Cytotoxicity against AZ521 cells	Akihisa *et al*. (2013)
1-Benzoyl-1-detigloylohchinolal	AZ521 cells		IC_50_ = 81.6 µM	*In vitro*	Cytotoxicity against AZ521 cells	Akihisa *et al*. (2013)
Nimbolinin D	AZ521 cells		IC_50_ = 96.8 µM	*In vitro*	Cytotoxicity against AZ521 cells	Akihisa *et al*. (2013)
Trichilinin E	SGC-7901 cells		IC_50_ = 6.9 µM	*In vitro*	Cytotoxicity against SGC-7901 cells	Zhou *et al*. (2016)
**Lung cancer**						
Toosendanin		A549 cells, tumor xenograft model	50–400 nM	*In vitro and in vivo*	Enhancing apoptosis and inducing autophagy	Li *et al*. (2017)
Toosendanin		A549 and A549/DDP cells	50 μg/ml	*In vitro*	Inducing cell growth inhibition and enhancing CDDP sensitization	Zheng *et al*. (2018)
Toosendanin		A549 and H1975 cells	2–10 nM	*In vitro*	Inhibiting TGF-β1-induced EMT, adhesion, invasion, and migration	Luo *et al*. (2018)
Toosendanin		A549 cells	20–60 μM	*In vitro*	Inducing apoptosis	
12-Hydroxyamoorastatone		A549 cells	ED_50_ = 0.92 μg/ml	*In vitro*	Cytotoxicity against A549 cells	Ahn *et al*. (1994)
12-Hydroxyamoorastatin		A549 cells	ED_50_ = 0.04 μg/ml	*In vitro*	Cytotoxicity against A549 cells	Ahn et al. (1994)
12-Acetoxyamoorastatin		A549 cells	ED_50_ = 0.01 μg/ml	*In vitro*	Cytotoxicity against A549 cells	Ahn *et al*. (1994)
1,12-Diacetyltrichilin B		A549 cells	IC_50_ = 0.93 µM	*In vitro*	Cytotoxicity against A549 cells	Yuan *et al*. (2013)
Meliarachin C		A549 cells	IC_50_ = 63.6 µM	*In vitro*	Cytotoxicity against A549 cells	Akihisa *et al*. (2013)
1-*O*-Decinnamoyl-1-*O*-benzoylohchinin		A549 cells	IC_50_ = 82.3 µM	*In vitro*	Cytotoxicity against A549 cells	Akihisa *et al*. (2013)
1-*O*-Decinnamoyl-1-*O*-benzoyl-23-hydroxyohchininolide		A549 cells	IC_50_ = 90.1 µM	*In vitro*	Cytotoxicity against A549 cells	Akihisa *et al*. (2013)
1-Benzoyl-1-detigloylohchinolal		A549 cells	IC_50_ = 83.5 µM	*In vitro*	Cytotoxicity against A549 cells	Akihisa *et al*. (2013)
**Colorectal cancer**						
Toosendanin	CRC SW480 cells		0.5 µM	*In vitro*	Inducing apoptosis	Wang *et al*. (2015)
Toosendanin	HEK293T, SW620, HCT-15/5FU-R cells and BALB/c nude mice		1–2 μM; 0.69 mg/kg for 22 days/i.p	*In vitro and in vivo*	Inhibiting cell proliferation and inducing apoptosis	Wang *et al*. (2020b)
12-Hydroxyamoorastatone	HCT-15 cells		ED_50_ = 1.6 μg/ml	*In vitro*	Cytotoxicity against HCT-15 cells	Ahn *et al*. (1994)
12-Hydroxyamoorastatin	HCT-15 cells		ED_50_ = 0.08 μg/ml	*In vitro*	Cytotoxicity against HCT-15 cells	Ahn *et al*. (1994)
12-Acetoxyamoorastatin	HCT-15 cells		ED_50_ = 0.04 μg/ml	*In vitro*	Cytotoxicity against HCT-15 cells	Ahn *et al*. (1994)
1,12-Diacetyltrichilin B	SW480 cells		IC_50_ = 0.04 µM	*In vitro*	Cytotoxicity against SW480 cells	Yuan *et al*. (2013)
**Glioblastoma**
Toosendanin	GBM U87 and C6 cell lines		10 nM	*In vitro*	Inhibiting cell proliferation and inducing apoptosis	Cao *et al*. (2016)
Toosendanin	U-138MG		50–150 nM	*In vitro*	Inducing apoptosis	Wang *et al*. (2020c)
**Leukemia**
Toosendanin	HL-60 cells		12.5–100 nM	*In vitro*	Inducing apoptosis	Ju *et al*. (2012)
Toosendanin	K562 cells		30, 50 nM	*In vitro*	Inhibiting cell proliferation and inducing apoptosis	
Toosendanin	HL-60 cells		20–80 ng/ml	*In vitro*	Inhibiting proliferation and cell cycle, inducing apoptosis	Ju *et al*. (2013)
Trichilinin B	HL-60 cells		IC_50_ = 22.2 µM	*In vitro*	Cytotoxicity against HL-60 cells	Pan *et al*. (2014b)
3-Deacetyl-28-oxosalannin	HL-60 cells		IC_50_ = 39.1 µM	*In vitro*	Cytotoxicity against HL-60 cells	Pan *et al*. (2014b)
3-Deacetyl-4′-demethyl-28-oxosalannin	HL-60 cells		IC_50_ = 2.8 µM	*In vitro*	Cytotoxicity against HL-60 cells	Pan *et al*. (2014b)
Ohchinin	HL-60 cells		IC_50_ = 56.3 µM	*In vitro*	Cytotoxicity against HL-60 cells	Pan *et al*. (2014b)
23-Hydroxyohchininolide	HL-60 cells		IC_50_ = 25.1 µM	*In vitro*	Cytotoxicity against HL-60 cells	Pan *et al*. (2014b)
21-Hydroxyisoohchininolide	HL-60 cells		IC_50_ = 22.7 µM	*In vitro*	Cytotoxicity against HL-60 cells	Pan *et al*. (2014b)
1,12-Diacetyltrichilin B	HL-60 cells		IC_50_ = 0.55 µM	*In vitro*	Cytotoxicity against HL-60 cells	Yuan *et al*. (2013)
Meliarachin C	HL-60 cells		IC_50_ = 0.65 µM	*In vitro*	Cytotoxicity against HL-60 cells	Yuan *et al*. (2013)
Toosendanin	HL-60 cells		IC_50_ = 0.005 µM	*In vitro*	Cytotoxicity against HL-60 cells	Yuan *et al*. (2013)
Meliarachin K	HL-60 cells		IC_50_ = 91.4 µM	*In vitro*	Cytotoxicity against HL-60 cells	Yuan *et al*. (2013)
Meliarachin G	HL-60 cells		IC_50_ = 82 µM	*In vitro*	Cytotoxicity against HL-60 cells	Yuan *et al*. (2013)
12-Dehydroneoazedarachin D	HL-60 cells		IC_50_ = 11.8 µM	*In vitro*	Cytotoxicity against HL-60 cells	Yuan *et al*. (2013)
Trichilinin D	HL-60 cells		IC_50_ = 61.2 µM	*In vitro*	Cytotoxicity against HL-60 cells	Yuan *et al*. (2013)
1-*O*-Cinnamoyltrichilinin	HL-60 cells		IC_50_ = 5.8 µM	*In vitro*	Cytotoxicity against HL-60 cells	Yuan *et al*. (2013)
Salannin	HL-60 cells		IC_50_ = 67.1 µM	*In vitro*	Cytotoxicity against HL-60 cells	Yuan *et al*. (2013)
Ohchinin	HL-60 cells		IC_50_ = 56.3 µM	*In vitro*	Cytotoxicity against HL-60 cells	Yuan *et al*. (2013)
Ohchinin–acetate	HL-60 cells		IC_50_ = 9.9 µM	*In vitro*	Cytotoxicity against HL-60 cells	Yuan *et al*. (2013)
1-*O*-Decinnamoyl-1-*O*-benzoylohchinin	HL-60 cells		IC_50_ = 54.8 µM	*In vitro*	Cytotoxicity against HL-60 cells	Yuan *et al*. (2013)
Ohchininolide	HL-60 cells		IC_50_ = 31.7 µM	*In vitro*	Cytotoxicity against HL-60 cells	Yuan *et al*. (2013)
1-*O*-Decinnamoyl-1-*O*-benzoylohchininolide	HL-60 cells		IC_50_ = 14.1 µM	*In vitro*	Cytotoxicity against HL-60 cells	Yuan *et al*. (2013)
23-Methoxyohchininolide A	HL-60 cells		IC_50_ = 4.9 µM	*In vitro*	Cytotoxicity against HL-60 cells	Yuan *et al*. (2013)
23-Hydroxyohchininolide	HL-60 cells		IC_50_ = 25.1 µM	*In vitro*	Cytotoxicity against HL-60 cells	Yuan *et al*. (2013)
1-*O*-Decinnamoyl-1-*O*-benzoyl-23-hydroxyohchininolide	HL-60 cells		IC_50_ = 12.6 µM	*In vitro*	Cytotoxicity against HL-60 cells	Yuan *et al*. (2013)
21-Hydroxyisoohchininolide	HL-60 cells		IC_50_ = 22.7 µM	*In vitro*	Cytotoxicity against HL-60 cells	Yuan *et al*. (2013)
17-Defurano-17-oxoohchinin	HL-60 cells		IC_50_ = 50.4 µM	*In vitro*	Cytotoxicity against HL-60 cells	Yuan *et al*. (2013)
1-*O*-Decinnamoyl-1-*O*-benzoyl-28-oxoohchinin	HL-60 cells		IC_50_ = 22.7 µM	*In vitro*	Cytotoxicity against HL-60 cells	Yuan *et al*. (2013)
Ohchinolal	HL-60 cells		IC_50_ = 39.2 µM	*In vitro*	Cytotoxicity against HL-60 cells	Yuan *et al*. (2013)
Mesendanin E	HL-60 cells		IC_50_ = 32.1 µM	*In vitro*	Cytotoxicity against HL-60 cells	Yuan *et al*. (2013)
1-Benzoyl-1-detigloylohchinolal	HL-60 cells		IC_50_ = 5.1 µM	*In vitro*	Cytotoxicity against HL-60 cells	Yuan *et al*. (2013)
1-Cinnamoyl-1-detigloylohchinolal	HL-60 cells		IC_50_ = 8.5 µM	*In vitro*	Cytotoxicity against HL-60 cells	Yuan *et al*. (2013)
Nimbolinin D	HL-60 cells		IC_50_ = 5.4 µM	*In vitro*	Cytotoxicity against HL-60 cells	Yuan *et al*. (2013)
**Ovarian cancer**						
Toosendanin	CAVO-3, SKOV-3 cells		500 nM	*In vitro*	Inhibiting invasion and migration	Li *et al*. (2018a)
Toosendanin	CAVO-3, ES-2 cells		500 nM	*In vitro*	Inducing apoptosis	Li *et al*. (2019)
Toosendanin	CAVO-3, A2870 cells		500 nM	*In vitro*	Inducing apoptosis	Li *et al*. (2018b)
12-Hydroxyamoorastatone	SKOV-3 cells		ED_50_ = 13.7 μg/ml	*In vitro*	Cytotoxicity against SKOV-3cells	Ahn *et al*. (1994)
12-Hydroxyamoorastatin	SKOV-3 cells		ED_50_ = 0.25 μg/ml	*In vitro*	Cytotoxicity against SKOV-3cells	Ahn *et al*. (1994)
12-Acetoxyamoorastatin	SKOV-3 cells		ED_50_ = 0.02 μg/ml	*In vitro*	Cytotoxicity against SKOV-3cells	Ahn *et al*. (1994)
**Hepatocellular carcinoma**						
Toosendanin	SMMC-7721 and Hep3B cells; BALB/c mice		IC_50_ = 0.5 µM; IC_50_ = 0.9 µM, 0.69 mg/kg/day for 15 days/i.p	*In vitro and in vivo*	Inducing apoptosis	He *et al*. (2010)
Toosendanin	SMMC-7721, Hep3B cells		0.5 μM	*In vitro*	Inhibiting cell proliferation and inducing apoptosis	Liu *et al*. (2016)
12-Ethoxynimbolinins G	SMMC-7721 cells		IC_50_ = 27.6 µM	*In vitro*	Cytotoxicity against SMMC-7721 cells	Zhang *et al*. (2018)
1,12-Diacetyltrichilin B	SMMC-7721 cells		IC_50_ = 0.36 µM	*In vitro*	Cytotoxicity against SMMC-7721 cells	Yuan *et al*. (2013)
Trichilinin E	HepG2 cells		IC_50_ = 6.9 µM	*In vitro*	Cytotoxicity against HepG2 cells	Zhou *et al*. (2016)
**Breast cancer**						
Toosendanin	MCF-7/ADM cells		50 nM; 0.69 mg/kg/every 2 days for 15 days/i.p	*In vitro and in vivo*	Inducing apoptosis	Wang *et al*. (2018)
Trichilinin B	SK-BR-3 cells		IC_50_ = 45.1 µM	*In vitro*	Cytotoxicity against SK-BR-3 cells	Pan *et al*. (2014b)
21-Hydroxyisoohchininolide	SK-BR-3 cells		IC_50_ = 91.5 µM	*In vitro*	Cytotoxicity against SK-BR-3 cells	Pan *et al*. (2014b)
Toosendansin H	MCF-7 cells		6.25 µM	*In vitro*	Cytotoxicity against MCF-7 cells	Li *et al*. (2020b)
Meliatoxin B1	MCF-7 cells		6.25 µM	*In vitro*	Cytotoxicity against MCF-7 cells	Li *et al*. (2020b)
1,12-Diacetyltrichilin B	MCF-7 cells		IC_50_ = 0.06 µM	*In vitro*	Cytotoxicity against MCF-7 cells	Yuan *et al*. (2013)
Meliarachin C	MCF-7 cells		IC_50_ = 90.4 µM	*In vitro*	Cytotoxicity against MCF-7 cells	Akihisa *et al*. (2013)
Trichilinin D	MCF-7 cells		IC_50_ = 41.5 µM	*In vitro*	Cytotoxicity against MCF-7 cells	Akihisa *et al*. (2013)
Salannin	MCF-7 cells		IC_50_ = 88.1 µM	*In vitro*	Cytotoxicity against MCF-7 cells	Akihisa *et al*. (2013)
1-*O*-Decinnamoyl-1-*O*-benzoylohchinin	MCF-7 cells		IC_50_ = 14.9 µM	*In vitro*	Cytotoxicity against MCF-7 cells	Akihisa *et al*. (2013)
1-*O*-Decinnamoyl-1-*O*-benzoylohchininolide	MCF-7 cells		IC_50_ = 54.5 µM	*In vitro*	Cytotoxicity against MCF-7 cells	Akihisa *et al*. (2013)
1-*O*-Decinnamoyl-1-*O*-benzoyl-23-hydroxyohchininolide	MCF-7 cells		IC_50_ = 4.3 µM	*In vitro*	Cytotoxicity against MCF-7 cells	Akihisa *et al*. (2013)
21-Hydroxyisoohchininolide	MCF-7 cells		IC_50_ = 91.5 µM	*In vitro*	Cytotoxicity against MCF-7 cells	Akihisa *et al*. (2013)
Ohchinolal	MCF-7 cells		IC_50_ = 94.4 µM	*In vitro*	Cytotoxicity against MCF-7 cells	Akihisa *et al*. (2013)
Mesendanin E	MCF-7 cells		IC_50_ = 70.5 µM	*In vitro*	Cytotoxicity against MCF-7 cells	Akihisa *et al*. (2013)
1-Benzoyl-1-detigloylohchinolal	MCF-7 cells		IC_50_ = 85.0 µM	*In vitro*	Cytotoxicity against MCF-7 cells	Akihisa *et al*. (2013)
1-Cinnamoyl-1-detigloylohchinolal	MCF-7 cells		IC_50_ = 94.8 µM	*In vitro*	Cytotoxicity against MCF-7 cells	Akihisa *et al*. (2013)
Nimbolinin D	MCF-7 cells		IC_50_ = 60.8 µM	*In vitro*	Cytotoxicity against MCF-7 cells	Akihisa *et al*. (2013)
**Ewing’s sarcoma**						
Toosendanin	SK-ES-1 cells		25, 50 µM	*In vitro*	Inducing apoptosis	Gao *et al*. (2019)
**Neuroma**						
Toosendanin	PC12 cells		0.87 µM	*In vitro*	Inducing apoptosis	Tang *et al*. (2004)
**Lymphoma**						
Toosendanin	U937 cells		0.87 µM	*In vitro*	Suppressing the cell cycle progression and inducing apoptosis	Zhang *et al*. (2005a)
12-Deacetyltrichilin I	P388 cells		IC_50_ = 0.011 μg/ml	*In vitro*	Cytotoxicity against P388 cells	Takeya *et al*. (1996b)
1-Acetyltrichilin H	P388 cells		IC_50_ = 0.47 µg/ml	*In vitro*	Cytotoxicity against P388 cells	Takeya *et al*. (1996b)
3-Deacetyltrichilin H	P388 cells		IC_50_ = 0.045 µg/ml	*In vitro*	Cytotoxicity against P388 cells	Takeya *et al*. (1996b)
1-Acetyl-3-deacetyltrichilin H	P388 cells		IC_50_ = 0.4 µg/ml	*In vitro*	Cytotoxicity against P388 cells	Takeya *et al*. (1996b)
1-Acetyl-2-deacetyltrichilin H	P388 cells		IC_50_ = 0.66 µg/ml	*In vitro*	Cytotoxicity against P388 cells	Takeya *et al*. (1996b)
Meliatoxin B1	P388 cells		IC_50_ = 5.4 µg/ml	*In vitro*	Cytotoxicity against P388 cells	Takeya *et al*. (1996b)
Trichilin H	P388 cells		IC_50_ = 0.16 µg/ml	*In vitro*	Cytotoxicity against P388 cells	Takeya *et al*. (1996b)
Trichilin D	P388 cells		IC_50_ = 0.055 µg/ml	*In vitro*	Cytotoxicity against P388 cells	Takeya *et al*. (1996b)
1,12-Diacetyltrichilin B	P388 cells		IC_50_ = 0.46 µg/ml	*In vitro*	Cytotoxicity against P388 cells	Takeya *et al*. (1996b)
**Pancreatic cancer**						
Toosendanin	PANC-1, AsPC-1 cells; BALB/c mice		200 nM; 0.2 mg/kg/day for 28 days/i.p	*In vitro and in vivo*	Inhibiting tumorous growth, inhibit migration and invasion, reversing EMT	Pei *et al*. (2017)
**Oral epithelial carcinoma**						
Trichilin H	KB cells		IC_50_ = 0.11 μg/ml	*In vitro*	Cytotoxicity against P388 cells	Tada *et al*. (1999)
Toosendanin	KB cells		IC_50_ = 3.82 μg/ml	*In vitro*	Cytotoxicity against P388 cells	Tada *et al*. (1999)
12-*O*-Methylvolkensin	KB cells		IC_50_ = 8.72 μg/ml	*In vitro*	Cytotoxicity against P388 cells	Tada *et al*. (1999)
**Cervical carcinoma**						
15-*O*-Deacetylnimbolidin B	HeLa S3 cells		IC_50_ = 0.1 µM	*In vitro*	Cytotoxicity against Hela S3 cells	Zhou *et al*. (2005)
12-*O*-Deacetyltrichilin H	HeLa S3 cells		IC_50_ = 0.48 µM	*In vitro*	Cytotoxicity against Hela S3 cells	Zhou *et al*. (2005)
15-*O*-Deacetyl-15-*O*-methylnimbolidin A	HeLa S3 cells		IC_50_ = 37.4 µM	*In vitro*	Cytotoxicity against Hela S3 cells	Zhou *et al*. (2005)
15-*O*-Deacetyl-15-*O*-methylnimbolidin B	HeLa S3 cells		IC_50_ = 28.3 µM	*In vitro*	Cytotoxicity against Hela S3 cells	Zhou *et al*. (2005)
**Melanoma**						
12-Hydroxyamoorastatone	SK-MEL-2 cells		IC_50_ = 0.38 μg/ml	*In vitro*	Cytotoxicity against SK-MEL-2 cells	Ahn *et al*. (1994)
12-Hydroxyamoorastatin	SK-MEL-2 cells		IC_50_ = 0.01 μg/ml	*In vitro*	Cytotoxicity against SK-MEL-2 cells	Ahn *et al*. (1994)
12-Acetoxyamoorastatin	SK-MEL-2 cells		IC_50_ = 0.0007 μg/ml	*In vitro*	Cytotoxicity against SK-MEL-2 cells	Ahn *et al*. (1994)
**Central nervous system tumors**						
12-Hydroxyamoorastatone	XF498 cells		IC_50_ = 2 μg/ml	*In vitro*	Cytotoxicity against XF498 cells	Ahn *et al*. (1994)
12-Hydroxyamoorastatin	XF498 cells		IC_50_ = 0.02 μg/ml	*In vitro*	Cytotoxicity against XF498 cells	Ahn *et al*. (1994)
12-Acetoxyamoorastatin	XF498 cells		IC_50_ = 0.007 μg/ml	*In vitro*	Cytotoxicity against XF498 cells	Ahn *et al*. (1994)
***Antifeeding and insecticide effects***						
Toosendanin	*M. separate*		LC_50_ = 252.23 μg/ml	*In vitro*	Causing the destruction of midgut epithelial cells, leading to the regurgitation, paralysis	Li *et al*. (2020a)
Toosendanin	*A. aegypti*		LC_50_ = 60.8 μg/ml	*In vitro*	Disrupting yolk deposition in oocytes, blood ingestion and digestion, and ovary ecdysteroid production	Ma *et al*. (2013)
Toosendanin	*B. plicatilis*		1.76–2.59 mg m^−3^	*In vitro*	Inhibiting digestive enzymes including pepsase and tryptase	Huang *et al*. (2017)
Toosendanin	*S. oryzae, C. ferrugineus*		LC_50_ = 675 and 875 ppm, respectively	*In vitro*	Reducing fecundity and expelling parasite	Xie *et al*. (1995)
Toosendanin	*S. mytilus*		LC_50_ = 6.4 μg/L	*In vitro*	Reducing the population density and fecundity	Xu *et al*. (2019)
Toosendanin	*P. rapae*		100–1,000 μg/ml	*In vitro*	Inducing antifeeding	
Toosendanin	*P. rapae*		3 μg/piece	*In vitro*	Reducing activity of microsomal multifunctional oxidase, protease, intestinal acetylase	Zhang and Zhao (1992a)
Toosendanin	*P. rapae*		400 ppm	*In vitro*	Destructing midgut tissue	Zhang and Zhao (1991)
Toosendanin	*P. rapae*		2 μg/piece	*In vitro*	Interfering physiological metabolism and inhibiting respiratory center	Zhang *et al*. (1992b)
Azedarachin A	*S. eridania*		MIC = 200 ppm	*In vitro*	Inducing antifeeding	Zhou *et al*. (1996)
Nimbolidin F	*S. eridania*		MIC = 500 ppm	*In vitro*	Inducing antifeeding	Zhou *et al*. (1997)
Ohchinolide C	*S. eridania*		MIC = 500 ppm	*In vitro*	Inducing antifeeding	Zhou *et al*. (1997)
Salannin	*S. eridania*		MIC = 500 ppm	*In vitro*	Inducing antifeeding	Zhou *et al*. (1997)
Nimbolidin C	*S. eridania*		MIC = 500 ppm	*In vitro*	Inducing antifeeding	Nakatani *et al*. (1996)
Nimbolidin D	*S. eridania*		MIC = 500 ppm	*In vitro*	Inducing antifeeding	Nakatani *et al*. (1996)
Nimbolidin E	*S. eridania*		MIC = 500 ppm	*In vitro*	Inducing antifeeding	Nakatani *et al*. (1996)
Nimbolidin B	*S. eridania*		MIC = 500 ppm	*In vitro*	Inducing antifeeding	Nakatani *et al*. (1996)
Toosendanin	*S. littoralis*		MIC = 200 ppm	*In vitro*	Inducing antifeeding	Nakatani *et al*. (2000)
Nimbolinin A	*S. littoralis*		MIC = 1,000 ppm	*In vitro*	Inducing antifeeding	Nakatani *et al*. (2000)
Nimbolinin C	*S. littoralis*		MIC = 1,000 ppm	*In vitro*	Inducing antifeeding	Nakatani *et al*. (2000)
Nimbolinin D	*S. littoralis*		MIC = 1,000 ppm	*In vitro*	Inducing antifeeding	Nakatani *et al*. (2000)
Nimbolinin B	*S. littoralis*		MIC = 1,000 ppm	*In vitro*	Inducing antifeeding	Nakatani *et al*. (2000)
Trichilinin D	*S. littoralis*		MIC = 1,000 ppm	*In vitro*	Inducing antifeeding	Nakatani *et al*. (2000)
Trichilinin E	*S. littoralis*		MIC = 1,000 ppm	*In vitro*	Inducing antifeeding	Nakatani *et al*. (2000)
1-*O*-Cinnamoyltrichilinin	*S. littoralis*		MIC = 1,000 ppm	*In vitro*	Inducing antifeeding	Nakatani *et al*. (2000)
1-Deoxy-3-tigloyl-11-methoxymeliacarpinin	*S. exigua*		3 μg/cm^2^	*In vitro*	Inducing antifeeding	Nakatani *et al*. (1993)
Trichilinin B	*S. eridania*		MIC = 1,000 ppm	*In vitro*	Inducing antifeeding	Nakatani (1999)
Trichilinin C	*S. eridania*		MIC = 1,000 ppm	*In vitro*	Inducing antifeeding	Nakatani (1999)
12-Hydroxyamoorastatin	*S. eridania*		MIC = 150 ppm	*In vitro*	Inducing antifeeding	Nakatani (1999)
Toosendanin	*S. eridania*		MIC = 300 ppm	*In vitro*	Inducing antifeeding	Nakatani (1999)
12-*O*-Acetylazedarachin A	*S. eridania*		MIC = 400 ppm	*In vitro*	Inducing antifeeding	Nakatani (1999)
13-*O*-Acetylazedarachin A	*S. littoralis*		MIC = 400 ppm	*In vitro*	Inducing antifeeding	Nakatani (1999)
Azedarachin B	*S. littoralis*		MIC = 200 ppm	*In vitro*	Inducing antifeeding	Nakatani (1999)
12-*O*-Acetylazedarachin B	*S. eridania*		MIC = 400 ppm	*In vitro*	Inducing antifeeding	Nakatani (1999)
Trichilin B	*S. eridania*		MIC = 200 ppm	*In vitro*	Inducing antifeeding	Nakatani (1999)
Trichilin H	*S. eridania*		MIC = 400 ppm	*In vitro*	Inducing antifeeding	Nakatani (1999)
Trichilin I	*S. eridania*		MIC = 400 ppm	*In vitro*	Inducing antifeeding	Nakatani (1999)
Trichilin J	*S. eridania*		MIC = 400 ppm	*In vitro*	Inducing antifeeding	Nakatani (1999)
Trichilin K	*S. eridania*		MIC = 400 ppm	*In vitro*	Inducing antifeeding	Nakatani (1999)
Trichilin L	*S. eridania*		MIC = 400 ppm	*In vitro*	Inducing antifeeding	Nakatani (1999)
1-Acetyltrichilin H	*S. littoralis*		MIC = 400 ppm	*In vitro*	Inducing antifeeding	Nakatani (1999)
12-Hydroxyamoorastatone	*S. eridania*		MIC = 250 ppm	*In vitro*	Inducing antifeeding	Nakatani (1999)
Isotoosendanin	*S. littoralis*		MIC = 300 ppm	*In vitro*	Inducing antifeeding	Nakatani (1999)
Neoazedarachin A	*S. littoralis*		MIC = 400 ppm	*In vitro*	Inducing antifeeding	Nakatani (1999)
Neoazedarachin B	*S. littoralis*		MIC = 400 ppm	*In vitro*	Inducing antifeeding	Nakatani (1999)
Neoazedarachin D	*S. littoralis*		MIC = 400 ppm	*In vitro*	Inducing antifeeding	Nakatani (1999)
1-Deacetylnimbolinin A	*S. littoralis*		MIC = 1,000 ppm	*In vitro*	Inducing antifeeding	Nakatani (1999)
2-Deacetylnimbolinin B	*S. littoralis*		MIC = 1,000 ppm	*In vitro*	Inducing antifeeding	Nakatani (1999)
Ohchinolide B	*S. eridania*		MIC = 1,000 ppm	*In vitro*	Inducing antifeeding	Nakatani (1999)
Ohchinolide C	*S. eridania*		MIC = 1,000 ppm	*In vitro*	Inducing antifeeding	Nakatani (1999)
3-*O*-Acetylohchinolal	*S. eridania*		MIC = 1,000 ppm	*In vitro*	Inducing antifeeding	Nakatani (1999)
Meliacarpinin A	*S. eridania*		MIC = 50 ppm	*In vitro*	Inducing antifeeding	Nakatani (1999)
Meliacarpinin C	*S. eridania*		MIC = 50 ppm	*In vitro*	Inducing antifeeding	Nakatani (1999)
Meliacarpinin D	*S. eridania*		MIC = 50 ppm	*In vitro*	Inducing antifeeding	Nakatani (1999)
Spirosendan	*S. littoralis*		MIC = 1,000 ppm	*In vitro*	Inducing antifeeding	Nakatani (1999)
1-Cinnamoyl-3-acetyl-11-hydroxymeliacarpin	*S. littoralis*		EC50 = 0.27 ppm	*In vitro*	Inducing antifeeding	Bohnenstengel *et al*. (1999)
Toosendanin	*P. rapae*		AFC50 = 0.21 mM	*In vitro*	Inducing antifeeding	Wang *et al*. (2020a)
Isotoosendanin	*P. rapae*		AFC_50_ = 0.46 mM	*In vitro*	Inducing antifeeding	Wang *et al*. (2020a)
12α-Hydroxyamoorastatone	*P. rapae*		AFC_50_ = 0.64 mM	*In vitro*	Inducing antifeeding	Wang *et al*. (2020a)
Mesendanin H	*P. rapae*		AFC_50_ = 0.11 mM	*In vitro*	Inducing antifeeding	Wang *et al*. (2020a)
Meliatoosenin E	*P. rapae*		AFC_50_ = 1.03 mM	*In vitro*	Inducing antifeeding	Wang *et al*. (2020a)
3-*O*-Acetylohchinolal	*P. rapae*		AFC_50_ = 0.89 mM	*In vitro*	Inducing antifeeding	Wang *et al*. (2020a)
Salannin	*P. rapae*		AFC_50_ = 1.35 mM	*In vitro*	Inducing antifeeding	Wang *et al*. (2020a)
Ohchinal	*P. rapae*		AFC_50_ = 1.79 mM	*In vitro*	Inducing antifeeding	Wang *et al*. (2020a)
12α-Hydroxyamoorastatone	*E. paenulata*		LD_50_ = 0.76 μg/cm^2^	*In vitro*	Inducing antifeeding and death	Carpinella *et al*. (2003)
Azadirachtin	*E. paenulata*		LD_50_ = 1.24 μg/cm^2^	*In vitro*	Inducing antifeeding and death	Carpinella *et al*. (2003)
Toosendanin	*E. paenulata*		ED_50_ = 3.69 μg/cm^2^	*In vitro*	Inducing antifeeding	Carpinella *et al*. (2003)
Azedarachin A	*S. exigua*		MIC = 200 ppm	*In vitro*	Inducing antifeeding	Huang *et al*. (1994)
12-*O*-Acetylazedarachin A	*S. exigua*		MIC = 400 ppm	*In vitro*	Inducing antifeeding	Huang *et al*. (1994)
***Anti-botulinum effect***						
Toosendanin	mice		9 mg/kg i.p	*In vivo*	Causing submicroscopic changes of presynaptic motor nerve endings	Xiong (1985)
Toosendanin	KM mice		8 mg/kg i.p	*In vivo*	Making the synaptosomes completely resistant to botulinum neurotoxin A-mediated cleavage of SNAP-25	Zhou *et al*. (2003)
Toosendanin	PC12 cells		35 μM	*In vitro*	Delaying channel formation and reducing the sizes of the channels	Li and Shi (2006)
Toosendanin	mice		10^–5^ g/ml	*In vivo*	Prolonging the paralysis time of botulism specimens	Li and Sun (1983)
Toosendanin	rats		8 mg/kg i.p	*In vivo*	Prolonging the paralysis time of botulism specimens	Li and Sun (1983)
Toosendanin	rats		7 mg/kg i.h	*In vivo*	Increasing the frequency of miniature end-plate potentials	Shih and Hsu (1983)
***Anti-inflammatory effects***						
1-*O*-Tigloyl-1-*O*-deacetyl-nimbolinin B	BV-2 cells		IC_50_ = 7.892 μM	*In vitro*	Inhibiting LPS-stimulated inflammatory via blocking the activation of NF-κB and JNK signaling pathways	Tao *et al*. (2014)
Trichilinin B	RAW 264.7 cells		IC_50_ = 29.2 μM	*In vitro*	Inhibiting LPS-stimulated inflammatory	Pan *et al*. (2014b)
3-Deacetyl-28-oxosalannolactone	RAW 264.7 cells		IC_50_ = 86 μM	*In vitro*	Inhibiting LPS-stimulated inflammatory	Pan *et al*. (2014b)
Ohchinin	RAW 264.7 cells		IC_50_ = 28.7 μM	*In vitro*	Inhibiting LPS-stimulated inflammatory	Pan *et al*. (2014b)
23-Hydroxyohchininolide	RAW 264.7 cells		IC_50_ = 58.6 μM	*In vitro*	Inhibiting LPS-stimulated inflammatory	Pan *et al*. (2014b)
21-Hydroxyisoohchininolide	RAW 264.7 cells		IC_50_ = 87.3 μM	*In vitro*	Inhibiting LPS-stimulated inflammatory	Pan *et al*. (2014b)
Toosendane B	RAW 264.7 cells		IC_50_ = 21.3 μM	*In vitro*	Inhibiting LPS-stimulated inflammatory	Hu *et al*. (2018)
Toosendane C	RAW 264.7 cells		IC_50_ = 20.7 μM	*In vitro*	Inhibiting LPS-stimulated inflammatory	Hu *et al*. (2018)
Ohchinin	RAW 264.7 cells		IC_50_ = 28.7 μM	*In vitro*	Inhibiting LPS-stimulated inflammatory	Akihisa *et al*. (2017)
Salannin	RAW 264.7 cells		IC_50_ = 27.9 μM	*In vitro*	Inhibiting LPS-stimulated inflammatory *via* reducing the expression of iNOS and COX-2	Akihisa *et al*. (2017)
Nimbolinin D	RAW 264.7 cells		IC_50_ = 24.4 μM	*In vitro*	Inhibiting LPS-stimulated inflammatory	Akihisa *et al*. (2017)
Mesendanin E	RAW 264.7 cells		IC_50_ = 23.8 μM	*In vitro*	Inhibiting LPS-stimulated inflammatory	Akihisa *et al*. (2017)
12-Ethoxynimbolinin A	HepG2 cells		10 μM	*In vitro*	Suppressing NF-κB activation	Zhu *et al*. (2014)
Nimbolinin C	HepG2 cells		10 μM	*In vitro*	Suppressing NF-κB activation	Zhu *et al*. (2014)
1-*O*-Tigloyl-1-*O*-debenzoylohchinal	HepG2 cells		10 μM	*In vitro*	Suppressing NF-κB activation	Zhu *et al*. (2014)
1-Acetyltrichilinin	HepG2 cells		10 μM	*In vitro*	Suppressing NF-κB activation	Zhu *et al*. (2014)
Trichilinin B	HepG2 cells		10 μM	*In vitro*	Suppressing NF-κB activation	Zhu *et al*. (2014)
1-*O*-Cinnamoyltrichilinin	HepG2 cells		10 μM	*In vitro*	Suppressing NF-κB activation	Zhu *et al*. (2014)
Trichilinin D	HepG2 cells		10 μM	*In vitro*	Suppressing NF-κB activation	Zhu *et al*. (2014)
Isotoosendanin	Mice		100 mg/kg	*In vivo*	Inhibiting acetic acid-induced vascular permeability and λ-carrageenan induced hind paw edema	Xie *et al*. (2008)
1-*O*-Tigloyl-1-*O*-debenzoylohchinal	Mice		100 mg/kg	*In vivo*	Inhibiting acetic acid-induced vascular permeability and λ-carrageenan induced hind paw edema	Xie *et al*. (2008)
Meliazedalides B	RAW 264.7		37.41 μM	*In vitro*	Inhibiting LPS-stimulated inflammatory	Qiu *et al*. (2019)
1-*O*-benzoyl-3-*O*-deactylnim-bolinin C	BV-2 cells		21.95 μM	*In vitro*	Inhibiting LPS-stimulated inflammatory	Park *et al*. (2020)
3-Deacetyl-12-*O*-methylvolkensin	BV-2 cells		21.37 μM	*In vitro*	Inhibiting LPS-stimulated inflammatory	Park *et al*. (2020)
12-*O*-Methyl-1-*O*-deacetyl-nimbolinin	BV-2 cells		23.16 μM	*In vitro*	Inhibiting LPS-stimulated inflammatory	Park *et al*. (2020)
Trichilinin B	BV-2 cells		15.28 μM	*In vitro*	Inhibiting LPS-stimulated inflammatory	Park *et al*. (2020)
1-*O*-Cinnamoyltrichilinin	BV-2 cells		7.73 μM	*In vitro*	Inhibiting LPS-stimulated inflammatory	Park *et al*. (2020)
1-Acetyltrichilinin	BV-2 cells		20.61 μM	*In vitro*	Inhibiting LPS-stimulated inflammatory	Park *et al*. (2020)
12*α*-Hydroxyamoorastatone	BV-2 cells		26.75 μM	*In vitro*	Inhibiting LPS-stimulated inflammatory	Park *et al*. (2020)
Toosendanin	Mice		0.5, 1.0 mg/kg i.p. for 7 days	*In vivo*	Alleviating DSS-induced colitis *via* inhibiting M1 macrophage polarization and regulating NLRP3 inflammasome and Nrf2/HO-1 signaling	Fan *et al*. (2019)
***Antibacterial and antifungal effects***						
1*α*,7*α*-Ditigloyloxy-3*α*-acetoxyl-12*α*-ethoxylnimbolinin	*P. gingivalis*		MIC = 15.2 μg/ml	*In vitro*	Inhibitory effects against *P. gingivalis*	Zhang *et al.* (2016)
1*α*-Tigloyloxy-3*α*-acetoxyl-7*α*-hydroxyl-12*β*-ethoxylnimbolinin	*P. gingivalis*		MIC = 31.25 μg/ml	*In vitro*	Inhibitory effects against *P. gingivalis*	Zhang *et al*. (2016)
12-Ethoxynimbolinins C	*P. gingivalis*		MIC = 15.6 μg/ml	*In vitro*	Inhibitory effects against *P. gingivalis*	Zhang *et al*. (2007)
1-*O*-Cinnamoyltrichilinin	*P. gingivalis*		MIC = 31.3 μg/ml	*In vitro*	Inhibitory effects against *P. gingivalis*	Zhang *et al*. (2007)
Trichilinin B	*P. gingivalis*		MIC = 31.5 μg/ml	*In vitro*	Inhibitory effects against *P. gingivalis*	Zhang *et al*. (2007)
7-Cinnamoyltoosendanin	*M. luteus*		MIC = 6.25 μg/ml	*In vitro*	Inhibitory effects against *M. luteus*	Liu *et al*. (2011)
7-Cinnamoyltoosendanin	*B. subtilis*		MIC = 25 μg/ml	*In vitro*	Inhibitory effects against *B. subtilis*	Liu *et al*. (2011)
Meliarachin D	*S. aureus*		MIC = 50 μg/ml	*In vitro*	Inhibitory effects against *S. aureus*	Su *et al*. (2011)
Meliarachin D	*B. subtilis*		MIC = 50 μg/ml	*In vitro*	Inhibitory effects against *B. subtilis*	Su *et al*. (2011)
Meliarachin H	*B. subtilis*		MIC = 25 μg/ml	*In vitro*	Inhibitory effects against *B. subtilis*	Su *et al*. (2011)

## Pharmacokinetics of Limonoids

There are few investigations on the pharmacokinetics of limonoids, and only several studies focus on the pharmacokinetics of TSN for elucidating the bioactivity and toxicity mechanism. In 2012, the first UPLC–MS/MS method was established for the pharmacokinetic determination of TSN in the rat plasma sample. The results showed that TSN possessed a fast absorption rate with a *T*_max_ (h) value of 0.63 h after oral administration. In terms of *V*_d_ level, oral administration (444,380.3 ± 204,747.7 ml/kg) had a wider distribution than intravenous administration (32,062.4 ± 18,562.8 ml/kg). Moreover, there was a significant variation of clearance between the two types of administration, and the results showed that TSN had a quick elimination rate for oral administration. However, the *C*_max_ values of TSN were found to be at a low level despite a high oral administration dose of 60 mg/kg body weight in rat. Furthermore, this study also found a low bioavailability of TSN (9.9%), which suggested that the bioactivity and toxicity of TSN may be related with its metabolites (Wang et al., 2013). In order to analyze metabolites, the human liver microsomes were incubated with TSN, and these metabolites of TSN were identified by ultra-high performance liquid chromatography–quadrupole-time of flight mass spectrometry. Six metabolites (M1–M6) were tentatively deduced by MS spectra information. Among them, M1, M2, and M3 were produced by oxidation of TSN, and M6 was produced by dehydrogenation of TSN, while M4 and M5 can be obtained by oxidation and dehydrogenation. It is worth noting that the stability of metabolites varied in terms of time, and M1–M5 possessed strong stability, which can last for 120 min. By contrast, M6 reached a maximum value at 20 min and then decreased gradually. These evidence provide powerful founding for unraveling the metabolic fate of TSN (Wu et al., 2013).

The reactive metabolites, which were formed by the bioactivation of drugs, can partially induce liver injury by causing protein dysfunction and DNA damage (Holt and Ju, 2006). According to reports, several compounds possessed apparent toxicity, which can be ascribed to the bioactivated furan ring (Druckova et al., 2007). Therefore, the TSN that contains a structural alert of the furan ring needs to be explored comprehensively for its metabolites and the bioactivation mechanism. One research showed that esterolysis and conjugation with amino acids were proved as the principal metabolic pathways of TSN. Further experiments found that CYP3A4 could bioactivate the furan ring and produce a *cis*-butene-1,4-dial intermediate, and the products possibly interacted with peptides or proteins, which might account for the hepatotoxicity caused by TSN. In addition, N-conjugation of the furan ring from diverse amino acids and glutathione (GSH), rather than the widely accepted S-conjugation, acted a key role in the elimination of reactive metabolites of TSN (Yu et al., 2014). Some studies also discussed the potential detoxification mechanism from the aspect of pharmacokinetics. After the combination use of *trans*-anethole, the *C*_max_ (294.8 ± 92.01 ng/L), AUC_(0–t)_ (347.2 ± 128.7 ng/Lh), AUC_(0–∞)_ (475.6 ± 210.8 ng/Lh), MRT_(0–t)_ (2.667 ± 0.989 h), and MRT_(0–∞)_ (5.386 ± 2.886 h) of TSN were decreased significantly, while the values of *V*_*z/F*_ (8.110 ± 5.502 L/kg) were notably increased. These outcomes indicated that co-administration of *trans*-anethole can decrease the absorption and bioavailability of TSN and shorten the elimination process of TSN, which reduced the risk of toxicity accumulation (Yu et al., 2020).

## Toxicity of Limonoids

Although limonoids, especially TSN, possess multiple biological activities, such as anti-tumor effect, insecticidal effect, anti-botulinum effect, anti-inflammation effect, *etc.*, a few toxic activities have also been discovered and gradually received attention (Table 3). *M. toosendan* and *M. azedarach* were recorded with mild toxicity in Chinese Pharmacopoeia. A large body of evidence found TSN as the main bioactive compound of these two herbs and is also responsible for their toxicity. *In vivo* and *in vitro* experiments have proven that hepatotoxicity, pregnancy-toxicity, and embryotoxicity were the primary toxic effects of TSN. In order to verify whether serum miRNAs can be used as potential indicators of hepatotoxicity induced by TSN, miRNA chip analysis was applied to detect the target miRNAs in serum. The experiment results showed that administration with TSN (10 mg/kg) could induce severe liver injury, which manifested as increased alanine aminotransferase/aspartate aminotransferase (ALT/AST) activity and typical pathological changes. Compared to the variation of ALT/AST activity, 22 potential miRNAs can better reflect the characteristics of liver toxicity induced by TSN. According to this research, the increased expression of miRNA-122-3p and mcmv-miRNA-m01-4-3p both existed in the TSN group and other hepatotoxicant groups, including acetaminophen, monocrotaline, and diosbuibin B. Interestingly, increased miRNA-367-3p was only found in the TSN group, which indicated that it could be used as a characteristic biomarker for discovering and discriminating TSN-induced liver injury (Yang et al., 2019).

**TABLE 3:** Toxicities and side effects of limonoids.

Compounds	Toxic type	Model	LD_50_/toxic dose range	Toxic reactions	Reference
Toosendanin	Pregnancy toxicity	Mice	0.2–0.8 mg/kg (i.p. for 7 days)	Increasing the level of IFN-γ, TNF-α, CD4^+^, and CD8^+^ T lymphocytes	Zhang *et al*. (2010a)
Toosendanin	Hepatotoxicity	Mice	10 mg/kg (i.p. for 6, 12, 24 h)	Increasing the level of serum ALT and AST and decreasing the expression of mir-367-3p	Yang *et al*. (2019)
Toosendanin	Hepatotoxicity	Mice	80 mg/kg (i.p. for 9 days)	Decreasing the body weight, increasing the level of serum ALT and AST, and enhancing the expression of Fmo3	Lu *et al*. (2016)
Toosendanin	Hepatotoxicity	Mice	3.75–15 mg/kg (i.p. for 24 h)	Decreasing the body weight, increasing the level of serum ALT and AST, and inducing energy metabolism disorder and hepatic steatosis	Yan *et al*. (2019)
Toosendanin	Hepatotoxicity	Primary hepatocyte cells	IC_50_ = 30.65 μM	Promoting Na^+^ influx and K^+^ efflux, decreasing the size of the cell membrane, and increasing the cell membrane permeability	Yan *et al*. (2019)
Toosendanin	Hepatotoxicity	Mice	10 mg/kg (i.p. for 6, 12, 24 h)	Increasing the level of serum ALT, AST, ALP, TBIL, ROS, and MDA and decreasing GSH content and Nrf2 expression	Jin *et al*. (2019b)
Toosendanin	Hepatotoxicity	L-02 cells	2–10 μM	Reducing GCL activity, GSH content, and expression of GCLC/GCLM and Nrf2; increasing ROS and Keap1 expression	Jin *et al*. (2019b)
Toosendanin	Hepatotoxicity	Primary hepatocyte cells	IC_50_ = 14.94 μM	Inducing mitochondrial dysfunction and caspase activation	Zhang *et al*. (2008)
Toosendanin	Embryotoxicity	Mice	0.46 mg/kg (i.p. for 3 days)	Inducing abortion	Zhang *et al*. (2005b)

Aside from investigating the role of miRNA expression alone, Lu *et al.* integrated the expression of microRNA with mRNA to explore the intricate and dynamic behavior of liver injury induced by TSN. An apparent liver injury was found 9 days after administration with TSN (80 mg/kg). Unexpectedly, the damage will be restored after 21 days of administration with TSN (80 mg/kg), which may account for liver regeneration. In addition, there existed dose- and time-specific alterations in global miRNA and mRNA expressions after being exposed to TSN. It can be inferred that the liver injury started with glutathione depletion, accompanied by mitochondrial dysfunction and lipid dysmetabolism, finally leading to hepatocyte necrosis in the liver from functional analyses of the miRNA–mRNA intersection dataset (Lu et al., 2016).

The application of multi-omics strategy, including proteomics and metabolomics, was considered as an advanced approach to explain the toxicity mechanism of TSN. Yan *et al.* found that triosephosphate isomerase 1 (TPI1) and α-enolase (ENOA), two glycolytic enzymes responsible for hepatotoxicity, were covalently modified by reactive metabolites of TSN. *In vitro* and *in vivo* experiments indicated that modifications would reduce the activity of TPI1 while inducing the activity of ENOA. Alterations of metabolites were also monitored and analyzed by metabolomics, and the outcomes showed a downward trend in tricarboxylic acid cycle, fatty acid β-oxidation, and amino acid metabolism, which revealed that the hepatocyte energy metabolism disorder could be induced by TSN (Yan et al., 2019). In another study, the role of Nrf2/GCL/GSH antioxidant signaling pathway in preventing hepatotoxicity from TSN was elucidated. It was found that TSN can decrease GSH content and Nrf2 expression, increase the expression of the Nrf2 inhibitor protein Kelch-like ECH-associated protein-1, and finally block the antioxidant signaling pathway to induce liver oxidative injury. Quercetin, a natural flavonoid with marked antioxidant ability, was validated to alleviate liver injury caused by TSN, which provided a new insight into compatibility detoxification of drugs (Jin Y. et al., 2019). In addition, fructus foeniculi, a typical detoxification drug for toosendan fructus, was reported to eliminate the toxicity of TSN by decreasing the absorption and bioavailability and accelerating the elimination process (Yu et al., 2020).

Since apoptosis plays an important role in inducing cell death, and TSN can exert its anti-tumor effect through the apoptotic pathway, whether apoptosis also acts as a critical regulator in liver damage caused by TSN needs further exploration. One representative study showed that TSN could inhibit primary rat hepatocyte growth with an IC_50_ value of 14.94 μM for 24 h. The underlying mechanism can be illustrated as TSN caused a decrease of mitochondrial membrane potential and intracellular ATP level and released cytochrome c to the cytoplasm, ultimately activating caspase-8, -9, and -3 to induce cell death. In the meantime, the activation of ROS and MAP kinases was also involved in this process (Zhang et al., 2008).

In recent years, with the increasing adverse effects of plant and vegetable pesticide residues on pregnant female individuals, the reproductive toxicity of TSN was also explored. The first study of reproductive toxicity caused by TSN was conducted by the team of Zhang, and they found that an intraperitoneal injection of TSN (0.46 mg/kg) could cause specific embryotoxicity to pregnant mice (Zhang X. F. et al., 2005). Later, in another study, the authors found that TSN-induced abortion was related with the imbalance of Th1/Th2 type cytokines, which manifested as increased levels of TNF-α and IFN-γ and CD4+/CD8+ ratio. These results partially proved that the immune imbalance is an important event in the reproductive toxicity induced by TSN (Zhang JL. et al., 2010).

The potential toxicity of limonoids has been noticed and emphasized in clinical application. Furthermore, the two herbs were commonly processed prior to clinical use, which can considerably reduce the toxicity. In addition, the compatibility of multiple herbs is another feasible method for toxicity reduction. In fact, countless innovative drugs, including atropine, scopolamine, and anisodamine, from Chinese medicinal herbs are toxic, but they are widely used for clinical purposes after an in-depth study. Therefore, the toxic limonoids (mainly focused on hepatotoxicity) could be candidate drugs *via* structural modification or targeted drug delivery.

## Conclusion

Taken together, the limonoids isolated from genus *Melia* possess certain similar skeleton structures and can be classified into diverse types, including trichilin class, vilasinin class, havanensin class, azadirone class, nimbolinin class, nimbolidin class, salannin class, ohchinolal class, meliacarpinin class, meliacarpin class, and others. Although enormous attempts have been devoted to synthesize, semi-synthesize, and biosynthesize the momentous TSN and other limonoids, no ideal tactic was established for the complicated synthesis process yet. Limonoids, which possess promising bioactivities, such as anti-tumor activity, antifeeding activity, anti-botulism effect, anti-inflammatory effect, and antibacterial effect, have obtained global acceptance in agricultural applications and contemporary medicine. In the meantime, the structure-related activity, toxic effects, and pharmacokinetics have also been summarized in this review. Notwithstanding that great breakthroughs have been made in the comprehensive exploration and application of limonoids, some in-depth works are still extremely urgent to be conducted in the future.

Firstly, as a marker of bioactivity and toxicity of *M. toosendan* and *M. azedarach*, TSN has a rigorous criterion for its content limit. Owing to the presence of a hemiacetal structure in the molecular structure, tautomerism will occur, which implies that there are two chromatographic peaks when detecting the content of TSN. In addition, the tautomerism also existed in 1-deacetylnimbolinin B, nimbolinin B, nimbolinin A, and 1-*O*-tigloyl-1-*O*-deacetyl-nimbolinin B (Shen et al., 2014). Even though the content of these compounds can be measured by calculating the sum of the areas of the two peaks, it is important to find another strategy to address this dilemma. Confused by this phenomenon, some researchers tried to utilize acetylation reactions to make 28-OH become a single compound. Nevertheless, this approach was difficult to achieve completely due to the small amount of acetic anhydride and pyridine and was not suitable for extracts of toosendan fructus and meliae cortex (Zhong et al., 1975). Since a tautomer may exert different roles in the treatment of diseases, even the opposite effect, it is therefore crucial to figure out whether there is any difference in bioactivity between the two tautomers.

Secondly, although diverse pharmacological activities of limonoids have been confirmed, their definite molecular targets are still unclear. Given the promising anti-tumor bioactivity against various types of cancers, more studies should be devoted to molecular and cellular mechanism research through the combination of metabolomics and proteomics. To date, there is a large body of research focusing on the pharmacological activities of limonoids by animal or *in vitro* cell models. However, few clinical trials have been exerted, which restricted its application. We have searched clinical trials of limonoids in “ClinicalTrials.gov” and “trialsearch.who.int”, but only one study was conducted to investigate the anti-hypercholesterolemic effect of a limonoid-rich beverage, and no results were posted. We acknowledge that it is a long journey from the discovery of natural bioactive compounds to the development of new drugs. Due to the reported side effect, the potential clinical trials of limonoids were limited. Nevertheless, cautious clinical trials are necessary after reasonable structural modification and toxicity evaluation.

Thirdly, an increasing number of studies reported the toxic effects of limonoids, and TSN has received the most attention. It was reported that hepatotoxicity was considered as the predominant toxicity, which is of great concern worldwide. Nevertheless, few studies focused on the toxicity of other limonoids that have a skeleton structure similar to that of TSN. Previous reports showed that the content of 1-deacetylnimbolinin B was higher than TSN in fructus toosendan, and the contents of nimbolinin B, nimbolinin A, and 1-*O*-tigloyl-1-*O*-deacetyl-nimbolinin B exhibited no noticeable difference with TSN (Shen et al., 2014). Thus, it is indispensable to carry out extensive toxicity screening of other limonoids for the comprehensive quality control of fructus toosendan and meliae cortex. Moreover, the lack of a clear toxicity mechanism seriously restricts its clinical application. Hence, the toxic molecular target and the metabolism changes *in vivo*, accompanied with potential detoxification and/or a toxicity enhancement metabolism pathway, need to be further explored. Interestingly, processing can reduce the toxicity of fructus toosendan, which suggests that heating, acidification, and other physical processes may transform toxic limonoids into less toxic compounds. Although the synthesis of toosendanin is challenging yet, a breakthrough has been made in the synthesis of azadirachtin (one limonoid of *genus* Azadirachta), which points out the direction of future study. The successful total synthesis was achieved by the strategy called “relay route” or “relay synthesis”, which attempted to degrade azadirachtin to a specific potential synthetic intermediate and then transform this back into the natural product. Therefore, a similar strategy is expected to be applied in the synthesis of toosendanin.

## Glossary

Cyt C: Cytochrome c
AIF: Apoptosis-inducing factor
Caspase: Cysteinyl aspartate-specific proteinase
Apaf-1: Apoptotic protease-activating factor-1
Bcl-2: B-cell lymphoma-2
PARP: Poly-ADP-ribose polymerase
TNFR: Tumor necrosis factor receptor
DR: Death receptor
FADD: Fas-associated death domain protein
DD: Death domain
DED: Death effector domain
DISC: Death-inducing signaling complex
TRAIL: Tumor necrosis factor (TNF)-related apoptosis-inducing ligand
DR5: Death receptor 5
CHOP: CCAAT/enhancer-binding protein homologous protein
ROS: Reactive oxygen species
JNK: c-Jun N-terminal kinase
miRNAs: MicroRNAs
EMT: Epithelial–mesenchymal transition
ECM: Extracellular matrix
TGF-β1: Transforming growth factor-β1
NSCLC: Non-small cell lung cancer
ADM: Adriamycin
SAR: Structure–activity relationships
DDS: Dextran sulfate sodium
NLRP3: NLR family pyrin domain containing 3
NO: Nitric oxide
TNF: Tumor necrosis factor
iNOS: Inducible nitric oxide synthase
COX-2: Cyclooxygenase
IL: Interleukin
LPS: Lipopolysaccharide
C/EBP-α: CCAAT/enhancer-binding proteins α
PPAR-γ: Peroxisome proliferator-activated receptor γ
HFD: High-fat diet
α-IFN: Alpha interferon
GSH: Glutathione
ALT: Alanine aminotransferase
AST: Aspartate aminotransferase
TPI1: Triosephosphate isomerase 1
ENOA: α-Enolase
Keap1: Kelch-like ECH-associated protein-1
